# Enhancing the Longevity and Structural Stability of Humidity Sensors: Iron Thin Films with Nitride Bonding Synthesized via Magnetic Field-Assisted Sparking Discharge

**DOI:** 10.3390/s24175466

**Published:** 2024-08-23

**Authors:** Stefan Ručman, Posak Tippo, Arisara Panthawan, Niwat Jhuntama, Nidchamon Jumrus, Pisith Singjai

**Affiliations:** Department of Physics and Materials Science, Faculty of Science, Chiang Mai University, Chiang Mai 50200, Thailand; posaktippo@gmail.com (P.T.); vampire_601@hotmail.com (A.P.); niwatjames@gmail.com (N.J.); nidchamon.j@hotmail.com (N.J.)

**Keywords:** sparking discharge, iron-based nanoparticles, nitrides, XPS, humidity sensors

## Abstract

Developing long-lasting humidity sensors is essential for sustainable advancements in nanotechnology. Prolonged exposure to high humidity can cause sensors to drift from their calibration points, leading to long-term accuracy issues. Our research aims to develop a fabrication method that produces stable sensors capable of withstanding the environmental challenges faced by humidity sensors. Traditional iron-based nanoparticles often require complex treatments, such as chemical modification or thermal annealing, to maintain their properties. This study introduces a novel, one-step synthesis method for iron-based thin films with exceptional stability. The synthesized films were thoroughly characterized using X-ray photoelectron spectroscopy (XPS) to evaluate their phase stability and nitride formation. The method proposed in this study employs an electrical sparking discharge process within a pure nitrogen atmosphere under a 0.2 T magnetic field, producing thin films composed of nanoparticles approximately 20 nm in size. The resulting films demonstrate superior performance in humidity sensing applications compared to conventional methods. This straightforward and efficient approach offers a promising path toward robust and sustainable humidity sensors.

## 1. Introduction

Humidity-dependent resistors, also known as humidity-sensitive resistors, are a type of humidity sensors, and they are electronic components that change their resistance in response to changes in humidity levels in the surrounding environment. These sensors are commonly used in various applications, including weather monitoring, HVAC systems, industrial processes, and consumer electronics. They are made of various materials, such as graphene flower/zinc oxide composites [1], two-dimensional heterojunction nanocomposites [2], novel coordination polymer and sodium metal ions [3], Zn^2+^-Doped TiO_2_:WO_3_ [4], magnesium silicate nanopowder [5], and many others. The choice of materials is dependent on the desired sensitivity, response time, operating range, environmental conditions, and cost considerations. The goal of our research was to create a material that will last long and be less susceptible to oxidation by water vapor. Creating a humidity sensor that can last long was our priority because we wanted to use a sustainable and easy-to-use nanomaterial fabrication process that can easily fabricate thin films. We used the sparking discharge process [6] in which tips of the wires are melted using electrical discharge, and thus fabricated nanoparticles are collected on the substrate. However, simply melting wires is not enough to ensure the longevity of the nanoparticles created. In our previous research [7], we created and fabricated highly-corrosion-resistant iron-based thin films by just sparking in a 0.2 T to 0.4 T magnetic field using a permanent magnet. Ref. [8] Chemical reactions in magnetic fields are widely known for their uncommon reactant products and crystalline structure [9]; in our experimental design, we used a magnetic field with the purpose of overcoming the need for thermal annealing, which, in turn, will make the process more sustainable and ecofriendly and additionally provide much needed longevity to thin films fabricated by sparking discharge. The use of magnetic fields in chemical reactions is well known for producing unique reactants, products, and crystalline structures [10,11]. The application of an external magnetic field during the laser ablation process enhances the crystallinity of the core–shell structure, increases the optical energy gap from 2.069 to 2.084 eV, and improves the nanoparticle concentration while reducing their size [11,12,13]. Inorganic molecule studies have shown that magnetic fields can affect crystallization in both thermal processing and solution chemistry. High magnetic fields can control material functionality, enhance phase transitions, and increase the coercivity of permanent magnets. Specifically, the iron phase in multicomponent crystalline alloys shows increased nucleation rates when annealed in magnetic fields. Additionally, the formation of aragonite over calcite under lower magnetic fields than theoretically required suggests that the magnetic field has a significant influence on the crystal structure [6,10,14].

## 2. Materials and Methods

**Figure 1:**
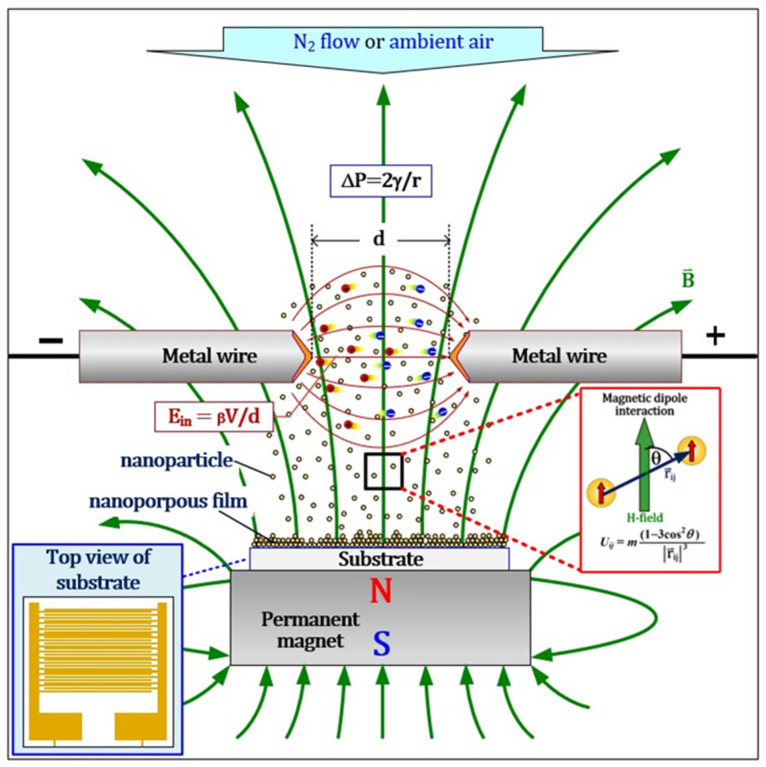
Schematic representation of chemical and fabrication process in our experiment.

**Figure 2:**
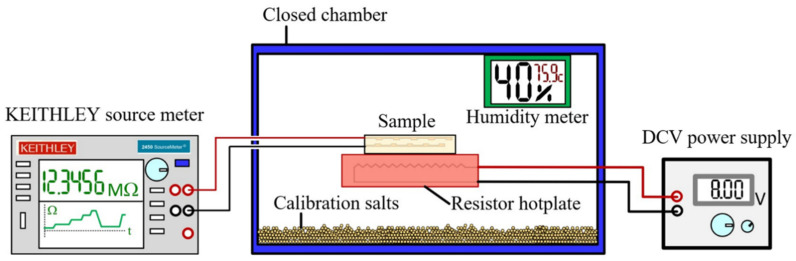
Experimental setup for humidity measurement at 90 °C; temperature was adjusted by using resistor on power supply 0.422 A and 10 V.

The sparking discharge generator was set according to previous research [10] and is depicted in Figure 1. On the magnet, a 5 × 50 μm ceramic interdigitated electrode IDE capacitor array sensor chip was placed on which nanoparticles were collected. For XPS analysis, samples are collected on quartz substrate, and characterization is carried out using XPS, Kratos Axis ULTRADLD (Kratos) spectrometer equipped with a monochromatic Al Kα X-ray source (1486.6 eV). The base pressure in the analysis chamber was approximately 5 × 10^−9^ torr. The X-ray source was used with an incidence angle of 45° to the surface plane. The operation was conducted at 150 W (15 kV and 10 mA) with a spot size of 700 × 300 μm^2^ and initial photo energy of 1.4 keV. The binding energy of the adventitious C 1s peak at 285 eV was used for calibration of wavelength shift. The spectra were acquired (at a constant take-off angle of 90°) with the pass energy of 20 eV and analyzed with the energy step of 0.1 eV using VISIONII (version 2.2.9) software. High-resolution transmission electron microscope (TEM) JEM 2010 was used with an accelerating voltage of 200 KV and a point resolution of 0.19 nm. Humidity measurements on different levels were obtained using different salts at 90 degrees Celsius and at room temperature in the setup, as depicted in Figure 2.

## 3. Results and Discussion

**Figure 3:**
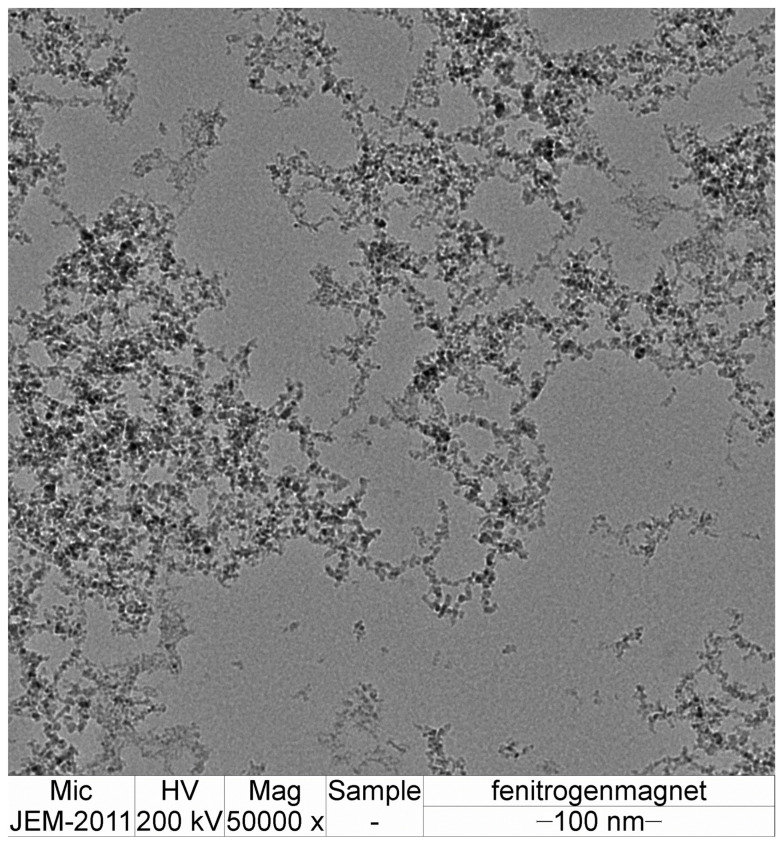
Higher magnification of nanoparticles prepared in nitrogen atmosphere under 0.2 T magnetic field.

**Figure 4:**
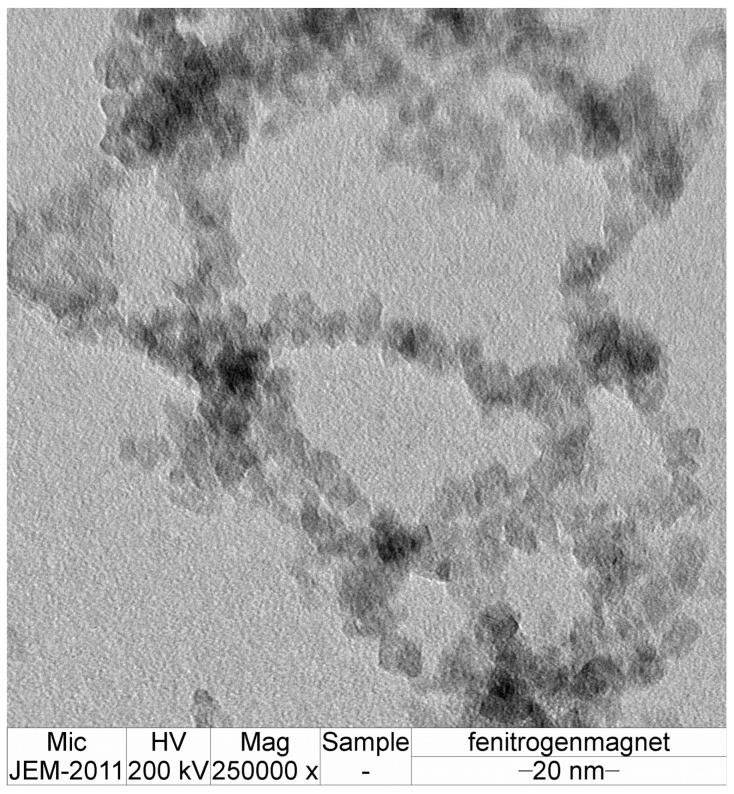
Close magnification of iron nanoparticles collected under magnetic field in nitrogen atmosphere.

Sparking discharge produces small nanoparticles that are deposited on the surface of a ceramic substrate. The primary particle size was determined using TEM. The distribution of nanoparticles is presented in Figure 3 and Figure 4.

The nanoparticles were deposited on the ceramic substrate for two hours, followed by the determination of humidity sensitivity based on different salts [15,16], as shown in the setup depicted in Figure 2. For the XPS analysis, particles were deposited on a glass substrate. The overall results are summarized in Table 1. Several conditions were made for the XPS analysis. Iron wires were sparked under eight conditions: in a nitrogen atmosphere; in a nitrogen atmosphere with a permanent 0.2 T magnet; in a nitrogen atmosphere with a permanent magnet treated with nitrates/nitrite solution; in an oxygen atmosphere with a permanent magnet; in a nitrogen environment without a magnet treated with a solution of nitrates/nitrites; and in an oxygen atmosphere without a magnetic field. See Table 1 for list of all conditions. These eight conditions were prepared on a glass substrate and left exposed for more than 120 days at room temperature and external conditions in a tropical climate in Chiang Mai, Thailand. Two additional samples were prepared fresh and immediately analyzed with XPS. These two conditions were put under a nitrogen atmosphere with a magnetic field and in an oxygen atmosphere with a magnetic field. We did not use fresh samples without a magnetic field because we previously described these XPS results in our previous work [7,17].

The Fe 2p3/2 peak is indeed stronger than the Fe 2p1/2 peak due to spin-orbit coupling [18]. The interaction between the electron’s spin and its orbital angular momentum leads to a slight energy difference between the two spin states (j = 3/2 and j = 1/2) within the same electron subshell (2p in this case). The Fe 2p3/2 state has four possible magnetic substates (mj = +3/2, +1/2, −1/2, and −3/2) due to its higher total angular momentum (j = 3/2). In contrast, the Fe 2p1/2 state only has two magnetic substates (mj = +1/2 and −1/2) because of its lower total angular momentum (j = 1/2) [19]. The Fe 2p3/2 state has twice the number of substates compared to Fe 2p1/2, so it can accommodate more electrons and thus gives rise to a stronger peak in the XPS spectrum. The typical binding energy range we found was between 710 eV and 712 eV, with some exceptions in certain conditions shifted toward zerovalent iron. However, the overall peaks belong to Fe^2+^ closer to 710 eV and Fe^3+^ around approximately 712 eV, as seen in previous studies [20,21,22]. Iron in the Fe^3+^ state lost one more electron compared to Fe^2+^. The removal of this additional electron can cause the remaining electrons to experience a stronger positive attraction from the nucleus, leading to slightly higher binding energies for the core electrons (reflected in the peaks at 712.0 eV and 713.0 eV). Freshly prepared sparked iron wire under a pure nitrogen atmosphere exhibited a shift toward 708.5 eV belonging to FeO; however, the presence of nitrogen at the 1s peak revealed the existence of an oxynitride state, as seen in previous work [23,24], and other states can be contributed, such as nitride (Me-N) peaks in the range of 395–398 eV. Pyridinic-N can show peaks around 398–400 eV. Nitriles (C≡N) can show peaks around 400–402 eV. Regarding nitro groups (NO_2_), nitrogen in nitro functional groups (-NO_2_) bonded to carbon typically has peaks between 405 and 407 eV [25,26,27]. We can observe from our samples that the presence of a pure nitrogen atmosphere during the preparation of thin films, as well as the presence or absence of a magnetic field, directly influences products of nitrification and results in different nitrogen 1s peaks. Iron prepared under a pure nitrogen atmosphere and under a magnetic field has the highest resistance toward corrosion, and we can see preserved peaks of nitride even after the treatment of the thin films with nitrite/nitrate solution.

Often, the O 1s peak can be deconvoluted into multiple peaks due to the presence of various oxygen species within the metal oxide. Lattice oxygen is oxygen bonded directly to the metal cations in the oxide structure and typically appears in the range of 529–532 eV [28]. Chemisorbed oxygen is oxygen molecules or atoms adsorbed on the metal oxide surface, which can show peaks at slightly higher binding energies (around 532–534 eV) [29]. Contamination from air exposure, such as water or CO_2_, can contribute to additional peaks at even higher binding energies (above 534 eV) [30].

**Figure 5:**
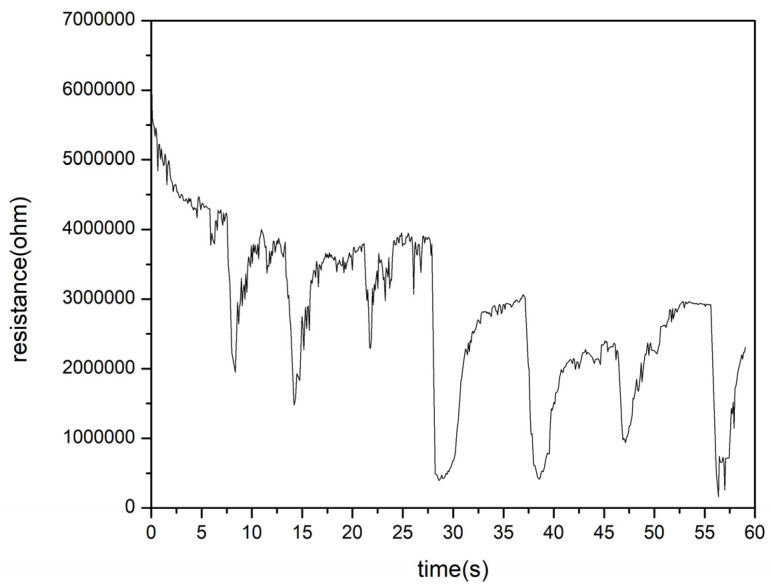
Responsivity of humidity sensor of sample aged for more than 6 months.

**Figure 6:**
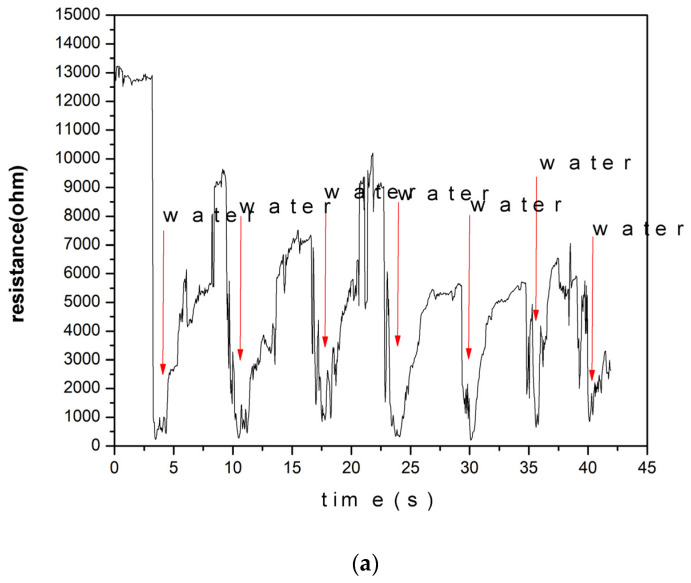

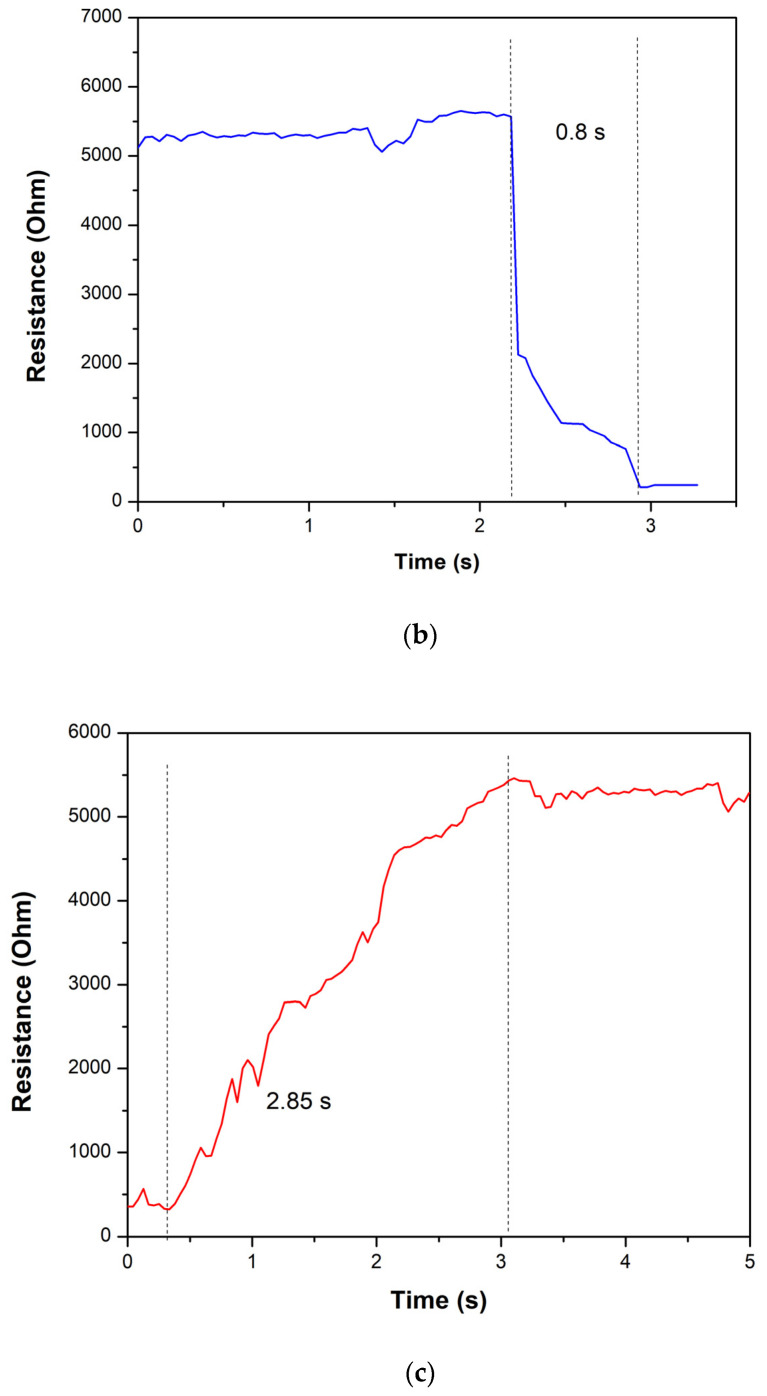
(**a**) Response and recovery times of humidity sensor aged for more than one year. (**b**) The response time of the humidity sensor aged for more than half a year. (**c**) The recovery time of the humidity sensor aged for more than half a year.

**Figure 7:**
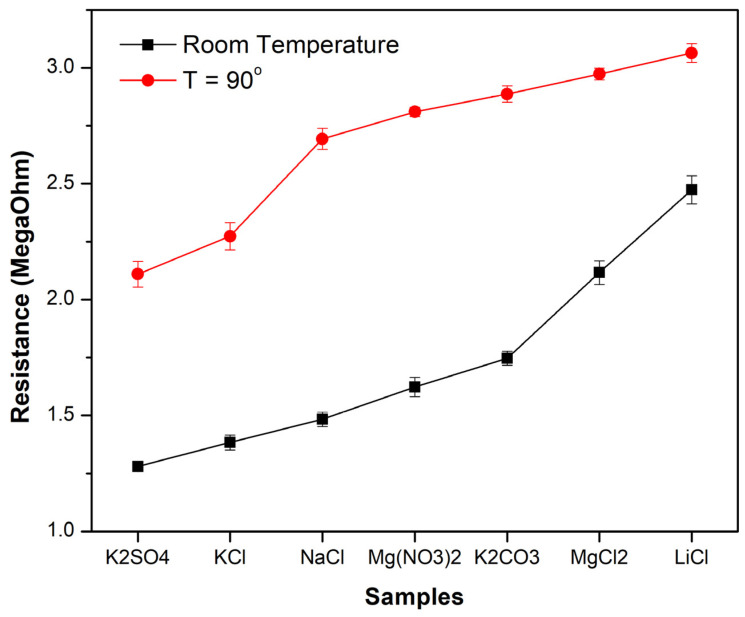
Relationship of resistivity of sensor with humidity concentration in box.

Based on the XPS data, most stability in terms of the obtained phases offered a condition where iron wires were sparked in a magnetic field under a total nitrogen atmosphere. This condition was further used to test the humidity sensing performance. A recovery measurement was taken after exposure to a spray of water, and the results of the responsivity test are presented in Figure 5. The response time was calculated to be 0.8 s, and the recovery time was 2.85 s based on the data in Figure 6. The resistance of the sensor prepared in the sparking of iron wire in a pure nitrogen atmosphere under a magnetic field, as a function of relative humidity, shows that the samples prepared under such a condition had a dynamic humidity response. This was tested at room temperature as well as at 90 degrees Celsius, as described in the setup shown in Figure 2 and the relationship presented in Figure 7. The following humidity concentrations were used to test the sensors at RT and at 90 degrees Celsius: K_2_SO_4_ (96%); KCl (89.3%); NaCl (75.6%); Mg(NO_3_)_2_ (51.4%); K_2_CO_3_ (43.16%); MgCl_2_ (32.8%); and LiCl (11.3%).

## 4. Conclusions

In our research, we addressed the convenience of production, miniaturization, sustained accuracy over time, and non-toxicity in the development of humidity sensors. By utilizing simple iron as the base material, we created a non-toxic foundation for a humidity sensor. The use of iron-based thin films synthesized through sparking discharge in a magnetic field has demonstrated exceptional stability and resistance to environmental degradation, which are crucial for ensuring the long-term accuracy and reliability of humidity sensors.

Through an X-ray photoelectron spectroscopy (XPS) analysis, we identified the conditions that reduce oxidation and enhance beneficial nitridation, which are essential for creating corrosion-resistant thin films. The response and recovery times of the aged sensor, shown in Figure 6b and Figure 6c, are 0.8 s and 2.85 s, respectively. These results, observed after more than a year, prove the effectiveness of our approach in achieving fast and reliable humidity sensing capabilities. In Table 2 different materials used in humidity sensing are presented and compared.

The synthesized iron-based thin films show increased resistance to oxidation by water vapor, a common issue that affects the longevity of traditional humidity sensors. The incorporation of a magnetic field during synthesis helps maintain the nitride phase, which is less prone to oxidation.

Additionally, the sparking discharge process employed in this study is sustainable and eco-friendly, eliminating the need for thermal annealing, reducing energy consumption, and simplifying the fabrication process. This sustainability aspect is vital for the widespread adoption and commercial viability of the sensors. Extensive characterization using techniques such as XPS confirmed the phase stability and nitride formation in the synthesized films, contributing to the sensor’s durability and consistent performance over extended periods.

## Figures and Tables

**Table 1:** XPS results of different conditions.

Substrate	C 1s	N 1s	O 1s	Fe 2p3/2
	BE (eV)	BE (eV)	BE (eV)	BE (eV)
Iron wires sparked under pure nitrogen atmosphere without magnetic field; aged for more than 120 days	284.861 (51.7%) 285.824 (22.2%) 286.995 (9.4%) 288.374 (16.8%)	396.145 (22.0%) 397.302 (9.6%) 398.839 (14.8%) 399.896 (21.2%) 402.077 (23.1%) 403.234 (9.3%)	529.839 (27.1%) 531.160 (21.8%) 532.029 (11.8%) 533.133 (30.0%) 534.526 (5.45%) 535.938 (3.9%)	709.787 (18.4%) 710.920 (26%) 712.336 (20.7%)
Iron sparked under nitrogen atmosphere under magnetic field of 0.2 T; aged for more than 120 days	284.943 (69.2%) 285.964 (11.8%) 286.908 (8.2%) 288.570 (10.8%)	396.322 (26.8%) 399.947 (31.0%) 402.275 (27.3%) 403.588 (14.9%)	530.012 (19.3%) 531.432 (19.4%) 532.762 (57.6%) 534.291 (3.7%)	710.119 (21.4%) 711.318 (25.7%) 712.734 (18.4%)
Iron sparked under nitrogen atmosphere with magnetic field and treatment with nitrite/nitrate 1% solution; aged for 120 days	284.898 (64.5%) 285.974 (16.5%) 287.032 (7.5%) 288.410 (11.5%)	399.896 (19.8%) 403.421 (17.0%) 407.079 (63.1%)	529.475 (11.8%) 530.841 (19.0%) 531.824 (26.2%) 532.644 (35.8%) 534.028 (4.5%) 535.776 (2.7%)	709.706 (22.2%) 710.936 (25.1%) 712.298 (17.5%)
Iron sparked under ambient oxygen atmosphere in the presence of magnetic field; aged for 120 days	284.937 (67.4%) 286.202 (14.6%) 287.033 (6.2%) 288.695 (11.9%)	400.097 (20.6%) 407.163 (79.4%)	530.129 (28.5%) 531.595 (35.3%) 532.807 (36.2%)	710.319 (20.1%) 711.329 (25.0%) 712.556 (19.3%)
Iron wires sparked in pure nitrogen atmosphere without magnetic field, treated with nitrite/nitrate 1% solution; aged for more than 120 days	285.007 (71.1%) 286.329 (12.4%) 287.367 (4.4%) 288.595 (12.2%)	396.458 (23.5%) 399.763 (36.7%) 402.588 (21.1%) 406.983 (18.7%)	530.029 (36.4%) 531.477 (38.6%) 532.906 (25.0%)	709.970 (21.2%) 711.071 (25.2%) 712.330 (18.6%)
Iron wire sparked under ambient oxygen atmosphere without magnetic field; aged for more than 120 days	285.037 (72.5%) 286.396 (14.9%) 287.397 (3.8%) 288.813 (8.8%)	400.211 (25.2%) 402.193 (14.0%) 407.101 (60.8%)	530.239 (18.2%) 531.714 (21.1%) 533.007 (58.1%) 534.427 (2.6%)	710.422 (22.2%) 711.586 (25.4%) 712.971 (17.8%)
Freshly prepared iron sparked under pure nitrogen atmosphere under magnetic field	284.975 (65.3%) 286.089 (20.7%) 287.033 (4.6%) 288.619 (9.4%)	396.245 (26.2%) 397.236 (11.3%) 399.186 (12.6%) 400.326 (8.9%) 402.325 (17.4%) 403.399 (10.4%) 406.868 (13.2%)	529.920 (46.0%) 531.341 (30.2%) 532.561 (14.6%) 533.435 (9.3%)	708.528 (3.4%) 710.164 (24.9%) 711.392 (21.6%) 712.682 (13.9%)
Freshly prepared iron sparked under ambient oxygen atmosphere under magnetic field	285.056 (64.9%) 286.264 (21.0%) 287.341 (3.5%) 288.681 (10.6%)	399.913 (27.2%) 406.935 (72.8%)	530.075 (31.2%) 531.240 (16.2%) 531.896 (13.9%) 533.025 (36.9%) 534.464 (1.8%)	710.138 (20.2%) 711.177 (26.1%) 712.530 (20.0%)

**Table 2:** Comparative performance of humidity sensors deposited using different methods.

Method	Material	Response Time (s)	Recovery Time (s)	Reference
sparking discharge	iron-based nanoparticles	0.8	2.85	This work
screen-printing method	graphene–carbon (G-C) ink	4	6	[31]
drop-casting method	GO film	0.3	0.3	[32]
screen-printing method	hexagonal-WO_3_ nanowires	1.5		[33]
electrostatic flocculation	hyaluronic acid (HA)-induced crumpling of Nb_2_CTx nanosheet	15.1	3.4	[34]
Hydrothermal method and green reducing agent route	graphene quantum dots (GQDs) and silver nanoparticles (AgNPs)	15	15	[35]

## Data Availability

The XPS data are available in the Supplementary Material.

## Supplementary Materials

The following supporting information can be downloaded at: https://www.mdpi.com/article/10.3390/s24175466/s1.
