# Supplementary material for: Enhancing the Longevity and Structural Stability of Humidity Sensors: Iron Thin Films with Nitride Bonding Synthesized via Magnetic Field-Assisted Sparking Discharge
Source: Sensors (Basel). 2024 Aug 23;24(17):5466. doi: 10.3390/s24175466 (PMC11397896; doi:10.3390/s24175466)

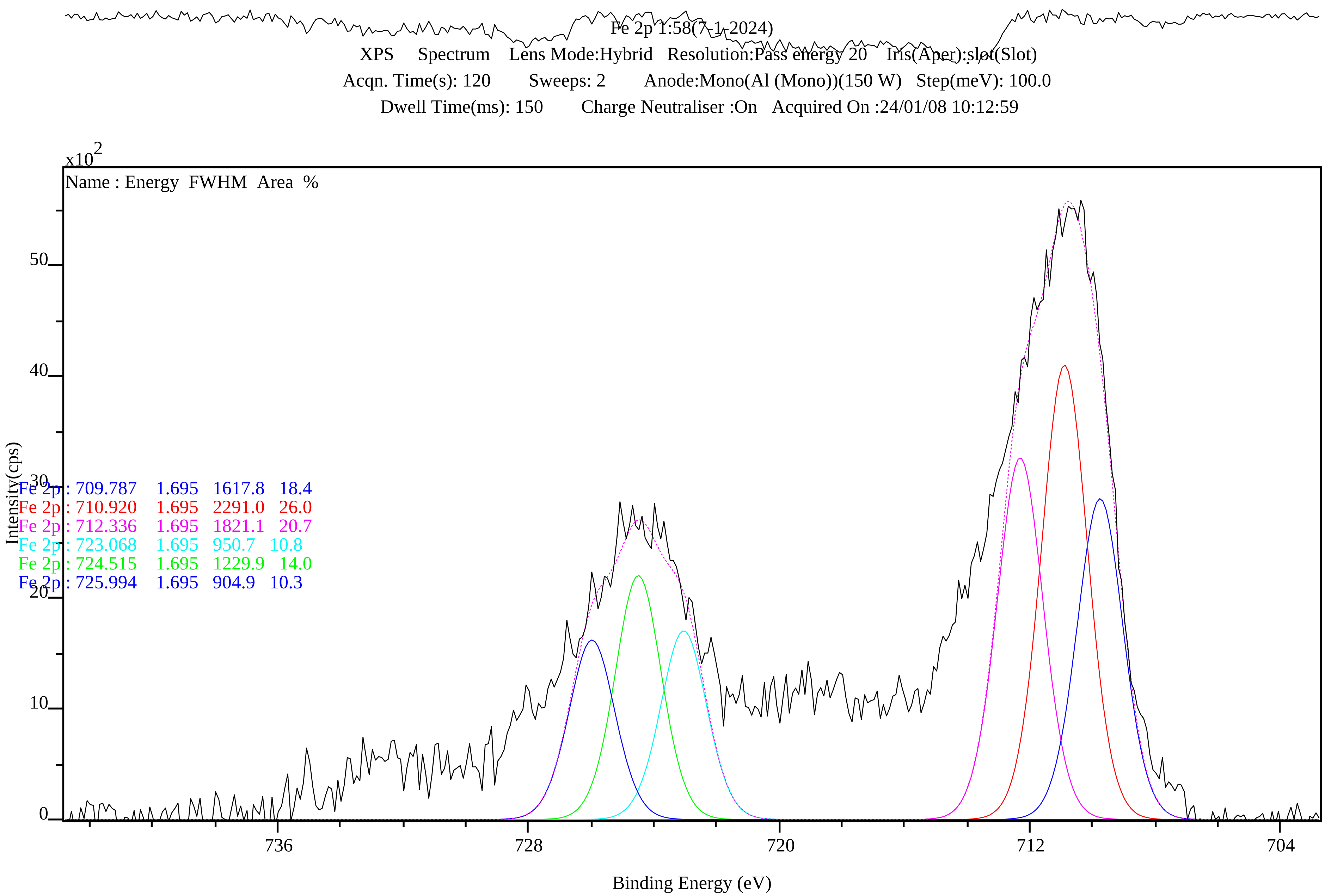

Fe 2p 1:58(7-1-2024)
XPS Spectrum Lens Mode:Hybrid Resolution:Pass energy 20 Iris(Aper):slot(Slot)
Acqn. Time(s): 120 Sweeps: 2 Anode:Mono(Al (Mono))(150 W) Step(meV): 100.0
Dwell Time(ms): 150 Charge Neutraliser :On Acquired On :24/01/08 10:12:59
x10^2
Name : Energy FWHM Area %
Fe 2p : 709.787 1.695 1617.8 18.4
Fe 2p : 710.920 1.695 2291.0 26.0
Fe 2p : 712.336 1.695 1821.1 20.7
Fe 2p : 723.068 1.695 950.7 10.8
Fe 2p : 724.515 1.695 1229.9 14.0
Fe 2p : 725.994 1.695 904.9 10.3
Intensity(cps)
50
40
30
20
10
0
736
728
720
712
704
Binding Energy (eV)

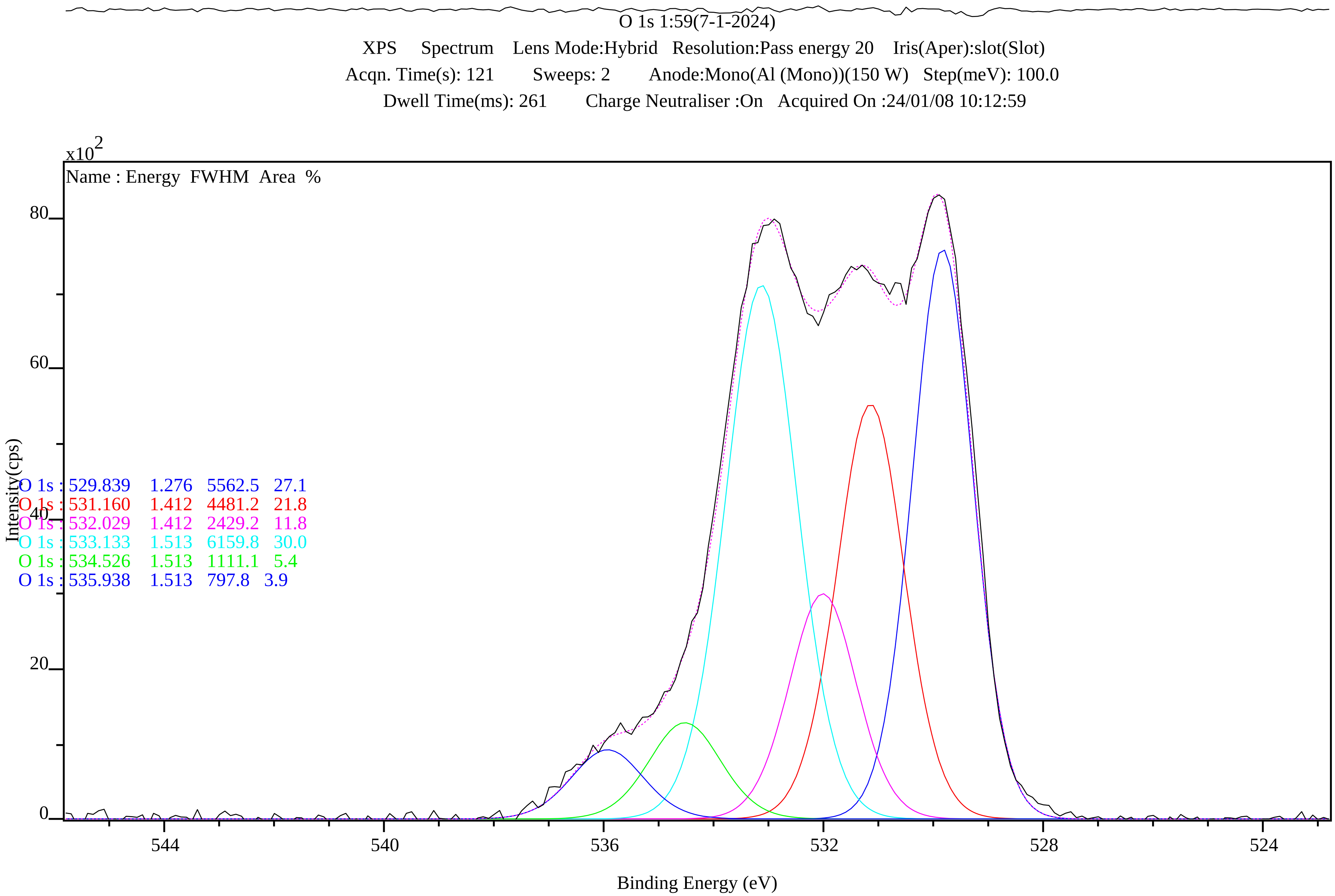
O 1s 1:59(7-1-2024)
XPS Spectrum Lens Mode:Hybrid Resolution:Pass energy 20 Iris(Aper):slot(Slot)
Acqn. Time(s): 121 Sweeps: 2 Anode:Mono(Al (Mono))(150 W) Step(meV): 100.0
Dwell Time(ms): 261 Charge Neutraliser :On Acquired On :24/01/08 10:12:59
x10^2
Name : Energy FWHM Area %
O 1s : 529.839 1.276 5562.5 27.1
O 1s : 531.160 1.412 4481.2 21.8
O 1s : 532.029 1.412 2429.2 11.8
O 1s : 533.133 1.513 6159.8 30.0
O 1s : 534.526 1.513 1111.1 5.4
O 1s : 535.938 1.513 797.8 3.9
Intensity(cps)
80
60
40
20
0
544
540
536
532
528
524
Binding Energy (eV)

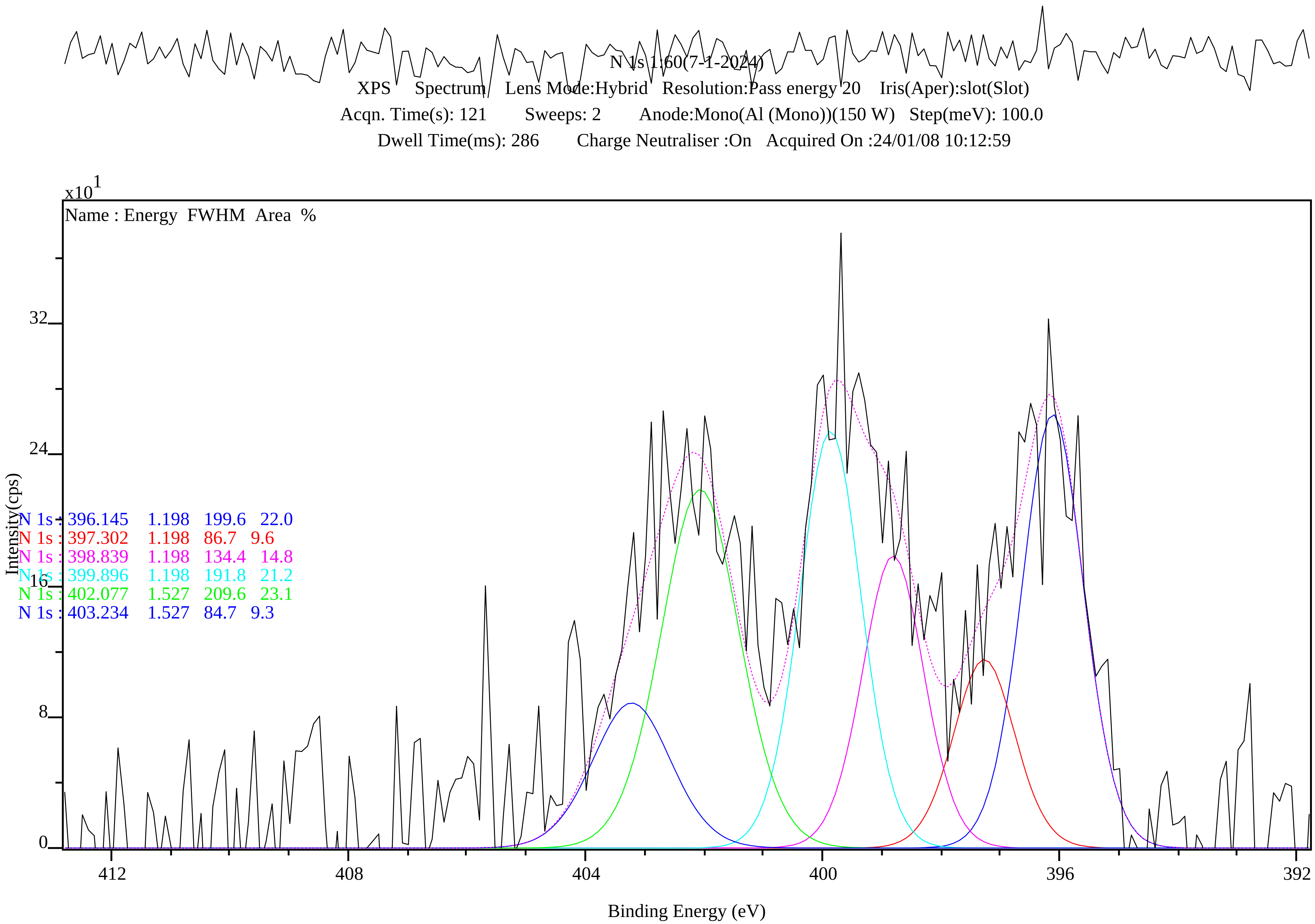
N 1s 1:60(7-1-2024)
XPS Spectrum Lens Mode:Hybrid Resolution:Pass energy 20 Iris(Aper):slot(Slot)
Acqn. Time(s): 121 Sweeps: 2 Anode:Mono(Al (Mono))(150 W) Step(meV): 100.0
Dwell Time(ms): 286 Charge Neutraliser :On Acquired On :24/01/08 10:12:59
x10^1
Name : Energy FWHM Area %
N 1s : 396.145 1.198 199.6 22.0
N 1s : 397.302 1.198 86.7 9.6
N 1s : 398.839 1.198 134.4 14.8
N 1s : 399.896 1.198 191.8 21.2
N 1s : 402.077 1.527 209.6 23.1
N 1s : 403.234 1.527 84.7 9.3
Intensity(cps)
32
24
16
8
0
412
408
404
400
396
392
Binding Energy (eV)

C 1s 1:61(7-1-2024)

XPS Spectrum Lens Mode:Hybrid Resolution:Pass energy 20 Iris(Aper):slot(Slot)

Acqn. Time(s): 120 Sweeps: 2 Anode:Mono(Al (Mono))(150 W) Step(meV): 100.0

Dwell Time(ms): 250 Charge Neutraliser :On Acquired On :24/01/08 10:12:59

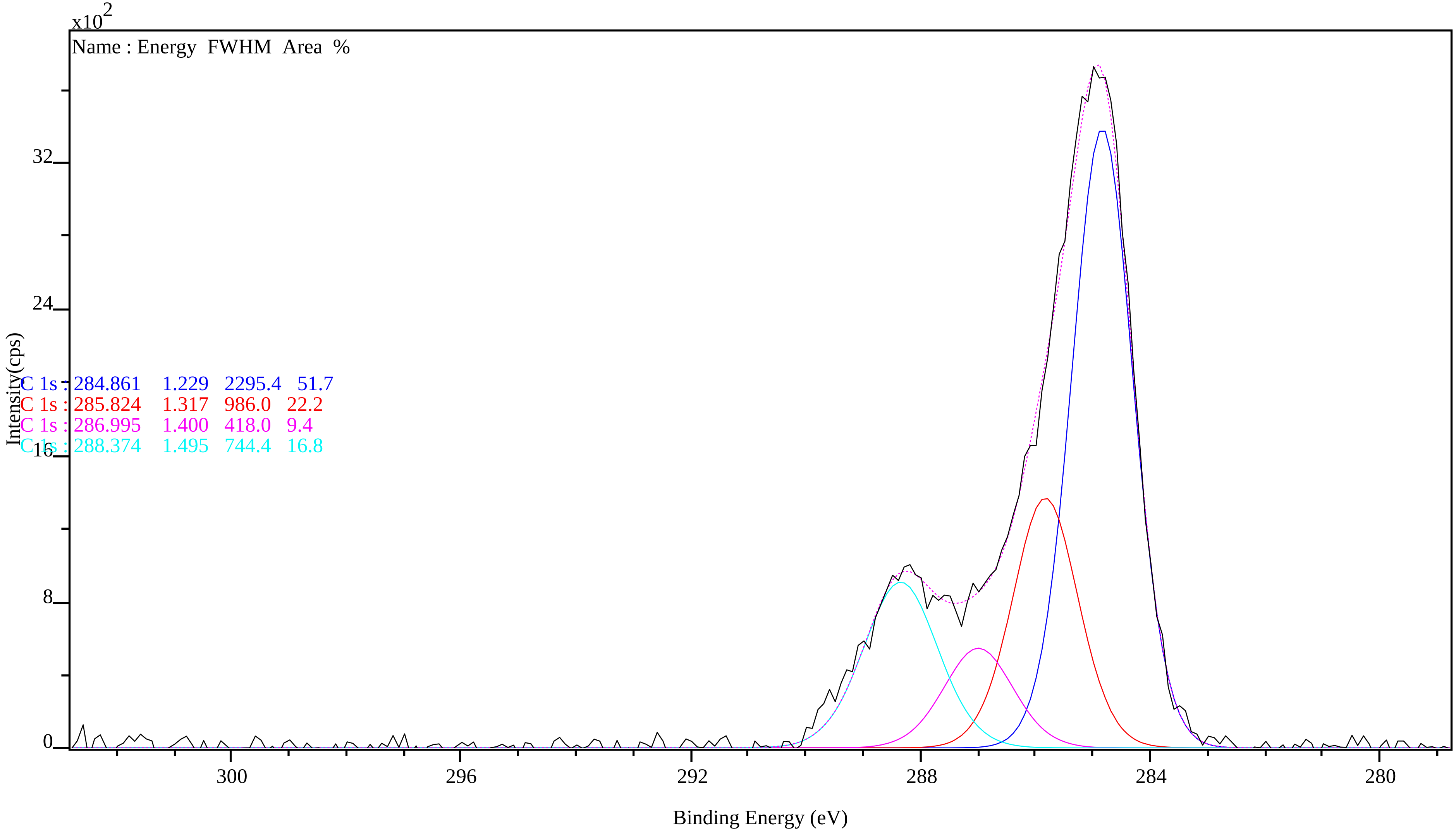

Supplement: Supplementary file 1 [file sensors-24-05466-s001.zip › XPS_7-1-2024/1.IRON N2/component.pdf]
