# Supplementary material for: Enhancing the Longevity and Structural Stability of Humidity Sensors: Iron Thin Films with Nitride Bonding Synthesized via Magnetic Field-Assisted Sparking Discharge
Source: Sensors (Basel). 2024 Aug 23;24(17):5466. doi: 10.3390/s24175466 (PMC11397896; doi:10.3390/s24175466)
Supplement: Supplementary file 1 [file sensors-24-05466-s001.zip › XPS_7-1-2024/1.IRON N2/report.pdf]

Quantification Report

/C=/data/7-1-2024/7-1-2024.dset Fri Jan 12 13:23:31 2024

State : Angle Name : Position 1

Peak	Type	Position BE (eV)	FWHM (eV)	Raw Area (cps eV)	RSF	Atomic Mass	Atomic Conc %	Mass Conc %
Fe 2p	Reg	711.000	5.432	451775.0	2.957	55.846	19.51	47.43
O 1s	Reg	530.000	5.019	240910.0	0.780	15.999	40.80	28.41
N 1s	Reg	396.000	7.808	12470.0	0.477	14.007	3.55	2.16
C 1s	Reg	285.000	2.647	62210.0	0.278	12.011	31.72	16.58
Si 2p	Reg	104.000	2.590	10620.0	0.328	28.086	4.43	5.41
