# Supplementary material for: Enhancing the Longevity and Structural Stability of Humidity Sensors: Iron Thin Films with Nitride Bonding Synthesized via Magnetic Field-Assisted Sparking Discharge
Source: Sensors (Basel). 2024 Aug 23;24(17):5466. doi: 10.3390/s24175466 (PMC11397896; doi:10.3390/s24175466)
Supplement: Supplementary file 1 [file sensors-24-05466-s001.zip › XPS_7-1-2024/2.IRON N2 magnet/component.pdf]

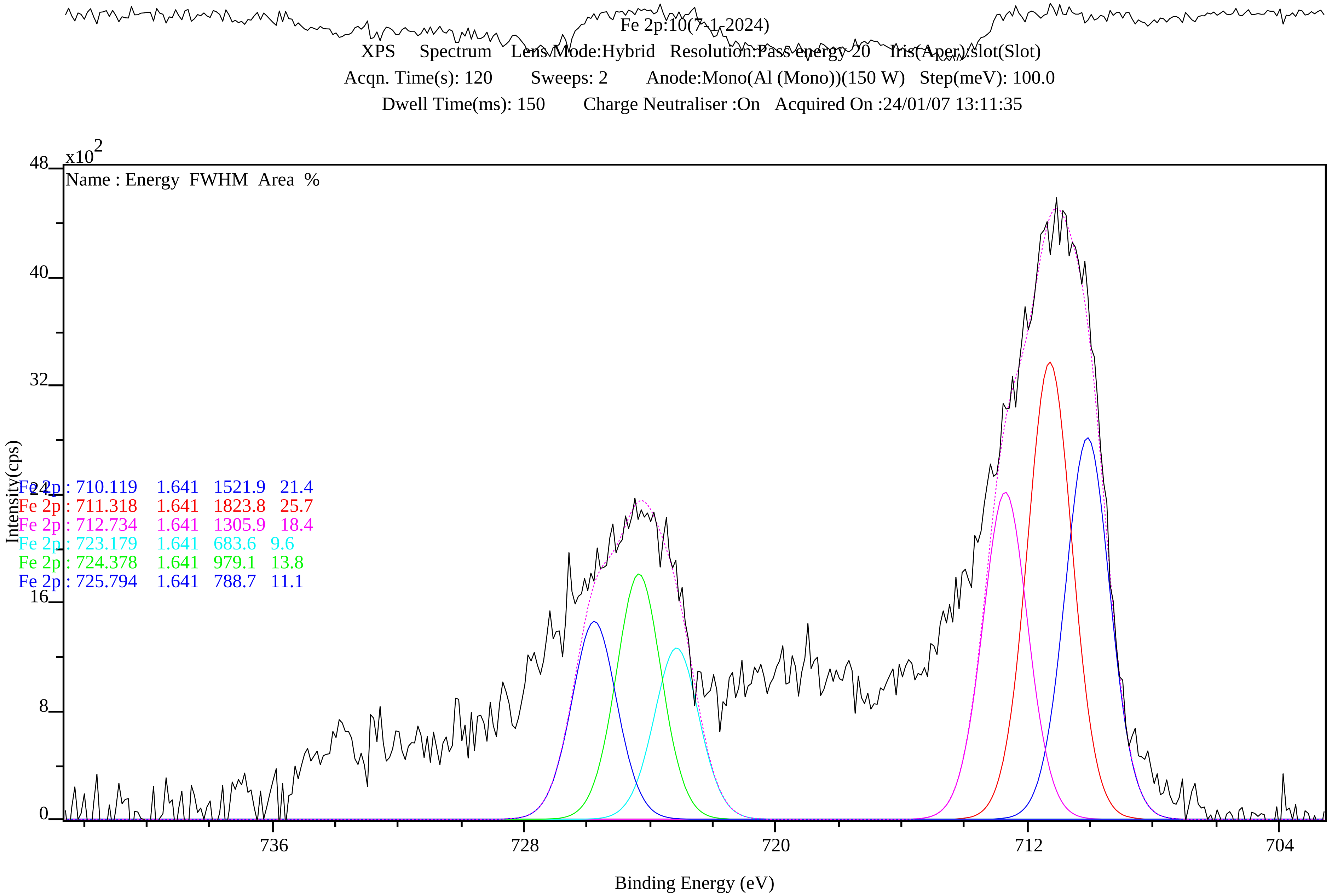
Fe 2p:10(7-1-2024)
XPS Spectrum Lens Mode:Hybrid Resolution:Pass energy 20 Iris(Aper):slot(Slot)
Acqn. Time(s): 120 Sweeps: 2 Anode:Mono(Al (Mono))(150 W) Step(meV): 100.0
Dwell Time(ms): 150 Charge Neutraliser :On Acquired On :24/01/07 13:11:35
x10^2
48
40
32
24
16
8
0
Name : Energy FWHM Area %
Fe 2p : 710.119 1.641 1521.9 21.4
Fe 2p : 711.318 1.641 1823.8 25.7
Fe 2p : 712.734 1.641 1305.9 18.4
Fe 2p : 723.179 1.641 683.6 9.6
Fe 2p : 724.378 1.641 979.1 13.8
Fe 2p : 725.794 1.641 788.7 11.1
Intensity(cps)
736
728
720
712
704
Binding Energy (eV)

O 1s:11(7-1-2024)

XPS Spectrum Lens Mode:Hybrid Resolution:Pass energy 20 Iris(Aper):slot(Slot)

Acqn. Time(s): 121 Sweeps: 2 Anode:Mono(Al (Mono))(150 W) Step(meV): 100.0

Dwell Time(ms): 261 Charge Neutraliser :On Acquired On :24/01/07 13:11:35

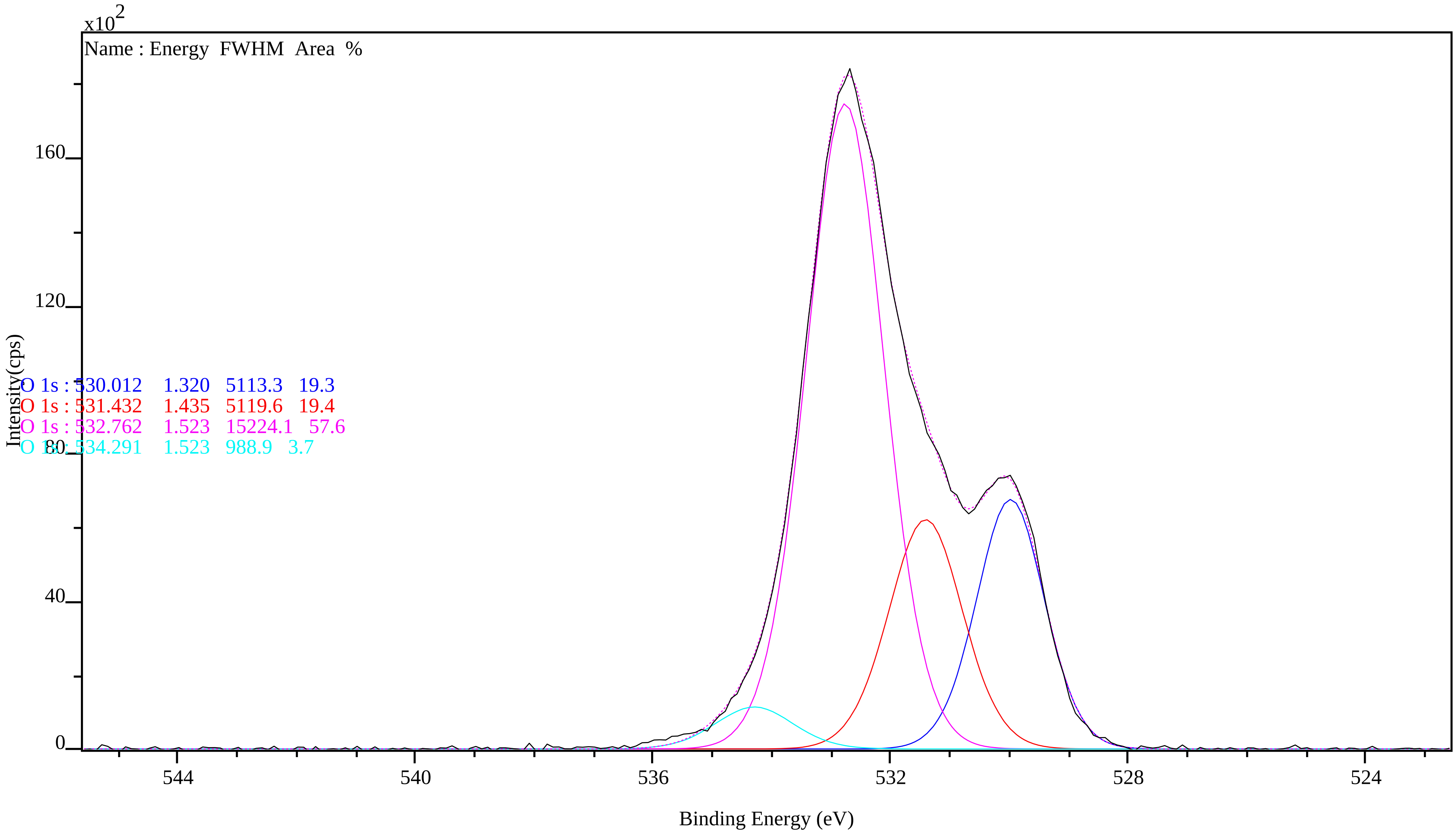

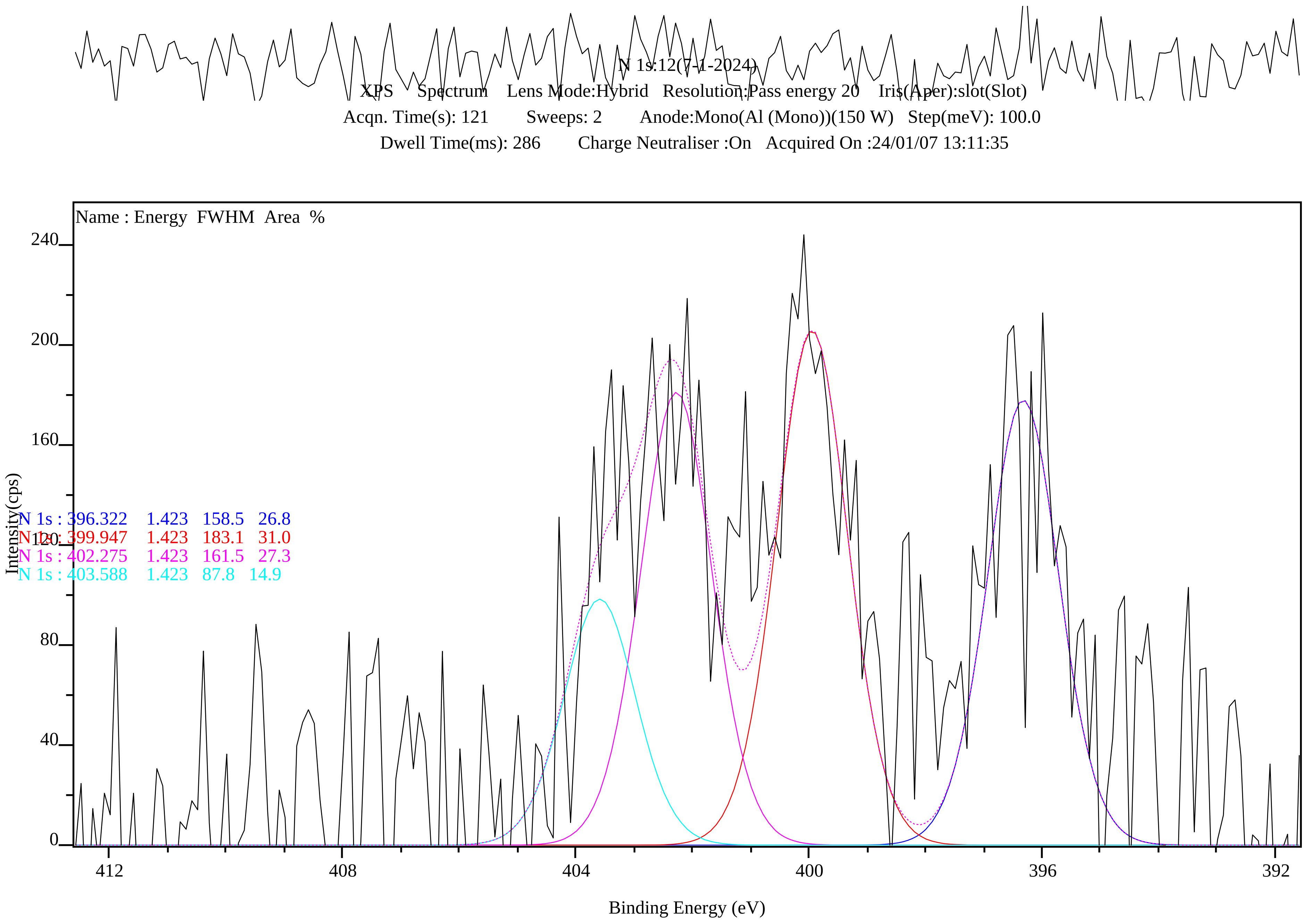
N 1s:12(7-1-2024)
XPS Spectrum Lens Mode:Hybrid Resolution:Pass energy 20 Iris(Aper):slot(Slot)
Acqn. Time(s): 121 Sweeps: 2 Anode:Mono(Al (Mono))(150 W) Step(meV): 100.0
Dwell Time(ms): 286 Charge Neutraliser :On Acquired On :24/01/07 13:11:35
Name : Energy FWHM Area %
N 1s : 396.322 1.423 158.5 26.8
N 1s : 399.947 1.423 183.1 31.0
N 1s : 402.275 1.423 161.5 27.3
N 1s : 403.588 1.423 87.8 14.9
Intensity(cps)
0
40
80
120
160
200
240
412
408
404
400
396
392
Binding Energy (eV)

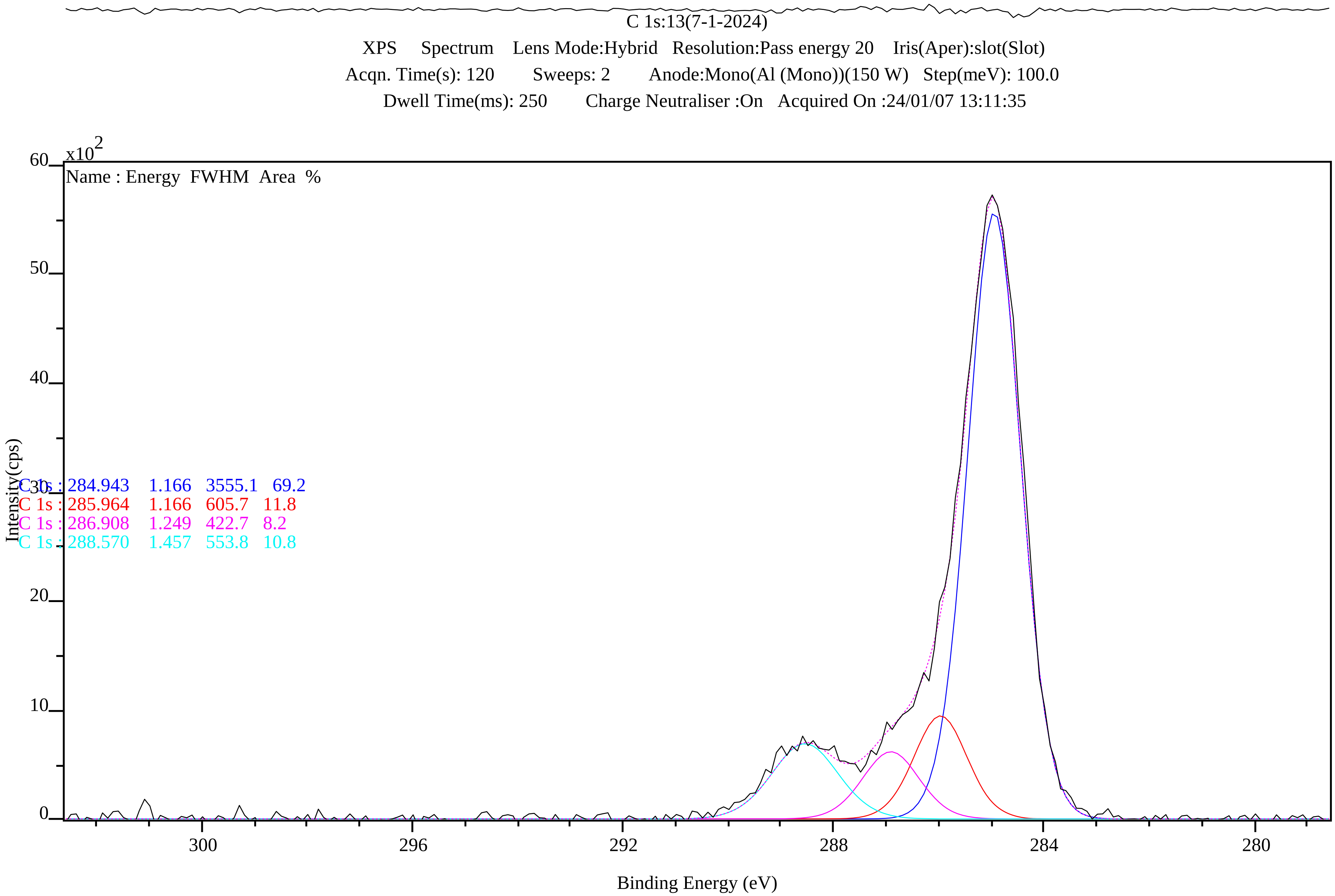

C 1s:13(7-1-2024)
XPS Spectrum Lens Mode:Hybrid Resolution:Pass energy 20 Iris(Aper):slot(Slot)
Acqn. Time(s): 120 Sweeps: 2 Anode:Mono(Al (Mono))(150 W) Step(meV): 100.0
Dwell Time(ms): 250 Charge Neutraliser :On Acquired On :24/01/07 13:11:35
x10^2
Name : Energy FWHM Area %
C 1s : 284.943 1.166 3555.1 69.2
C 1s : 285.964 1.166 605.7 11.8
C 1s : 286.908 1.249 422.7 8.2
C 1s : 288.570 1.457 553.8 10.8
Intensity(cps)
0
10
20
30
40
50
60
300
296
292
288
284
280
Binding Energy (eV)
