# Supplementary material for: Enhancing the Longevity and Structural Stability of Humidity Sensors: Iron Thin Films with Nitride Bonding Synthesized via Magnetic Field-Assisted Sparking Discharge
Source: Sensors (Basel). 2024 Aug 23;24(17):5466. doi: 10.3390/s24175466 (PMC11397896; doi:10.3390/s24175466)

wide:9(7-1-2024)

XPS Spectrum Lens Mode:Hybrid Resolution:Pass energy 80 Iris(Aper):slot(Slot)

Acqn. Time(s): 121 Sweeps: 1 Anode:Mono(Al (Mono))(150 W) Step(meV): 1000.0

Dwell Time(ms): 100 Charge Neutraliser :On Acquired On :24/01/07 13:08:38

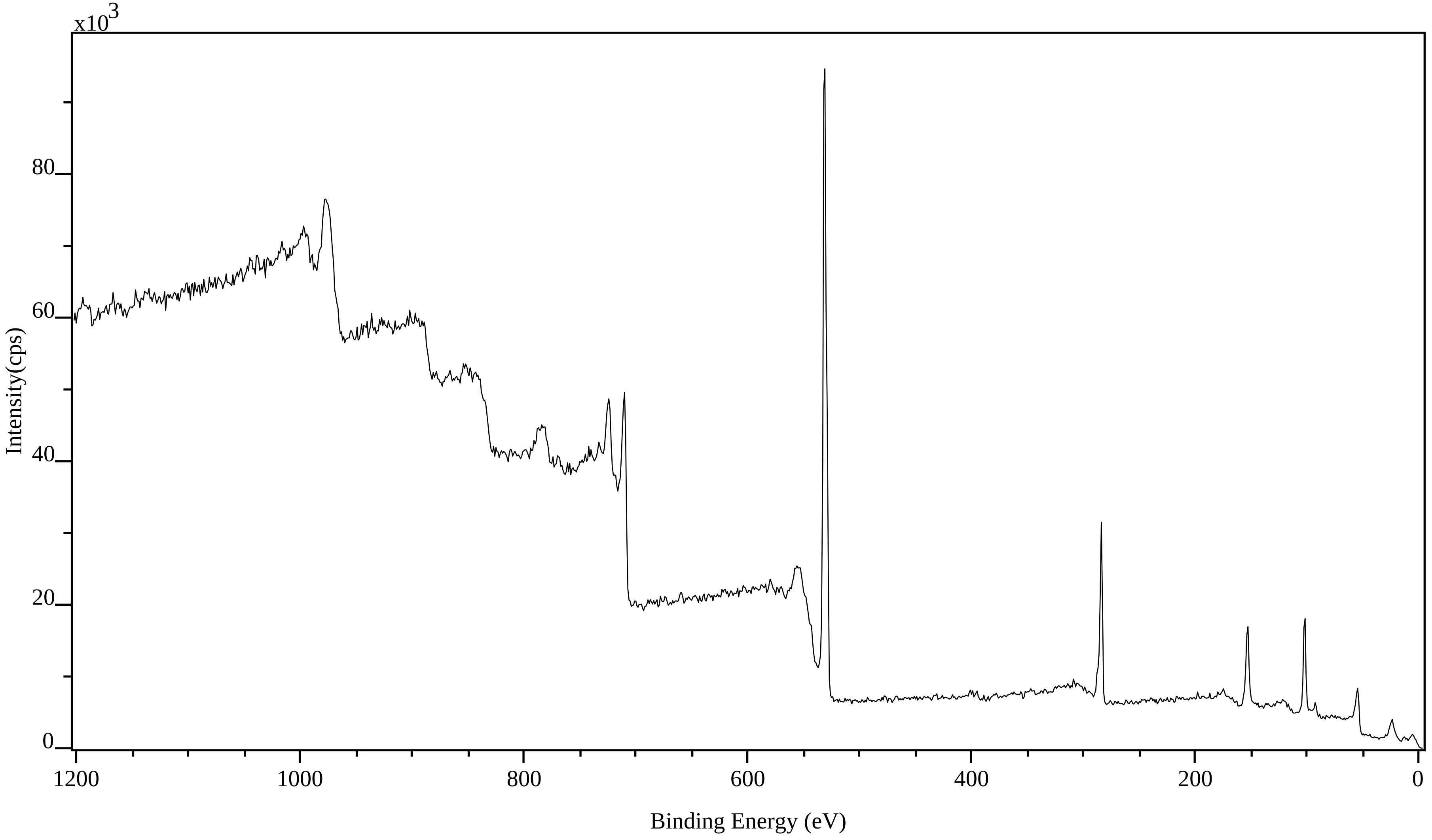

Supplement: Supplementary file 1 [file sensors-24-05466-s001.zip › XPS_7-1-2024/2.IRON N2 magnet/survey.pdf]
