# Supplementary material for: Enhancing the Longevity and Structural Stability of Humidity Sensors: Iron Thin Films with Nitride Bonding Synthesized via Magnetic Field-Assisted Sparking Discharge
Source: Sensors (Basel). 2024 Aug 23;24(17):5466. doi: 10.3390/s24175466 (PMC11397896; doi:10.3390/s24175466)
Supplement: Supplementary file 1 [file sensors-24-05466-s001.zip › XPS_7-1-2024/3.IRON N2 mag sample2/component.pdf]

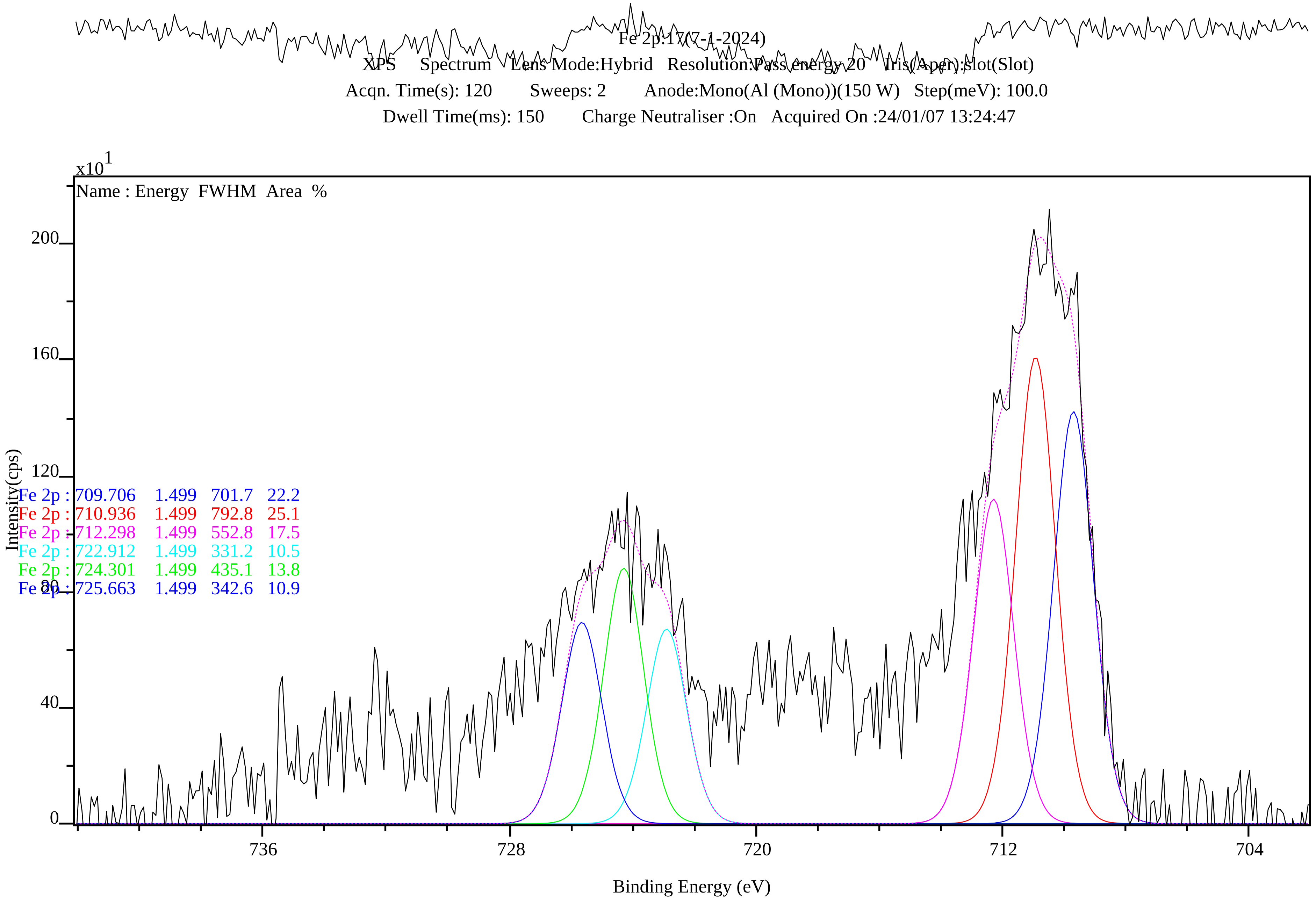
Fe 2p:17(7-1-2024)
XPS Spectrum Lens Mode:Hybrid Resolution:Pass energy 20 Iris(Aper):Slot(Slot)
Acqn. Time(s): 120 Sweeps: 2 Anode:Mono(Al (Mono))(150 W) Step(meV): 100.0
Dwell Time(ms): 150 Charge Neutraliser :On Acquired On :24/01/07 13:24:47
x10 1
Name : Energy FWHM Area %
Fe 2p : 709.706 1.499 701.7 22.2
Fe 2p : 710.936 1.499 792.8 25.1
Fe 2p : 712.298 1.499 552.8 17.5
Fe 2p : 722.912 1.499 331.2 10.5
Fe 2p : 724.301 1.499 435.1 13.8
Fe 2p : 725.663 1.499 342.6 10.9
Intensity(cps)
200
160
120
80
40
0
736
728
720
712
704
Binding Energy (eV)

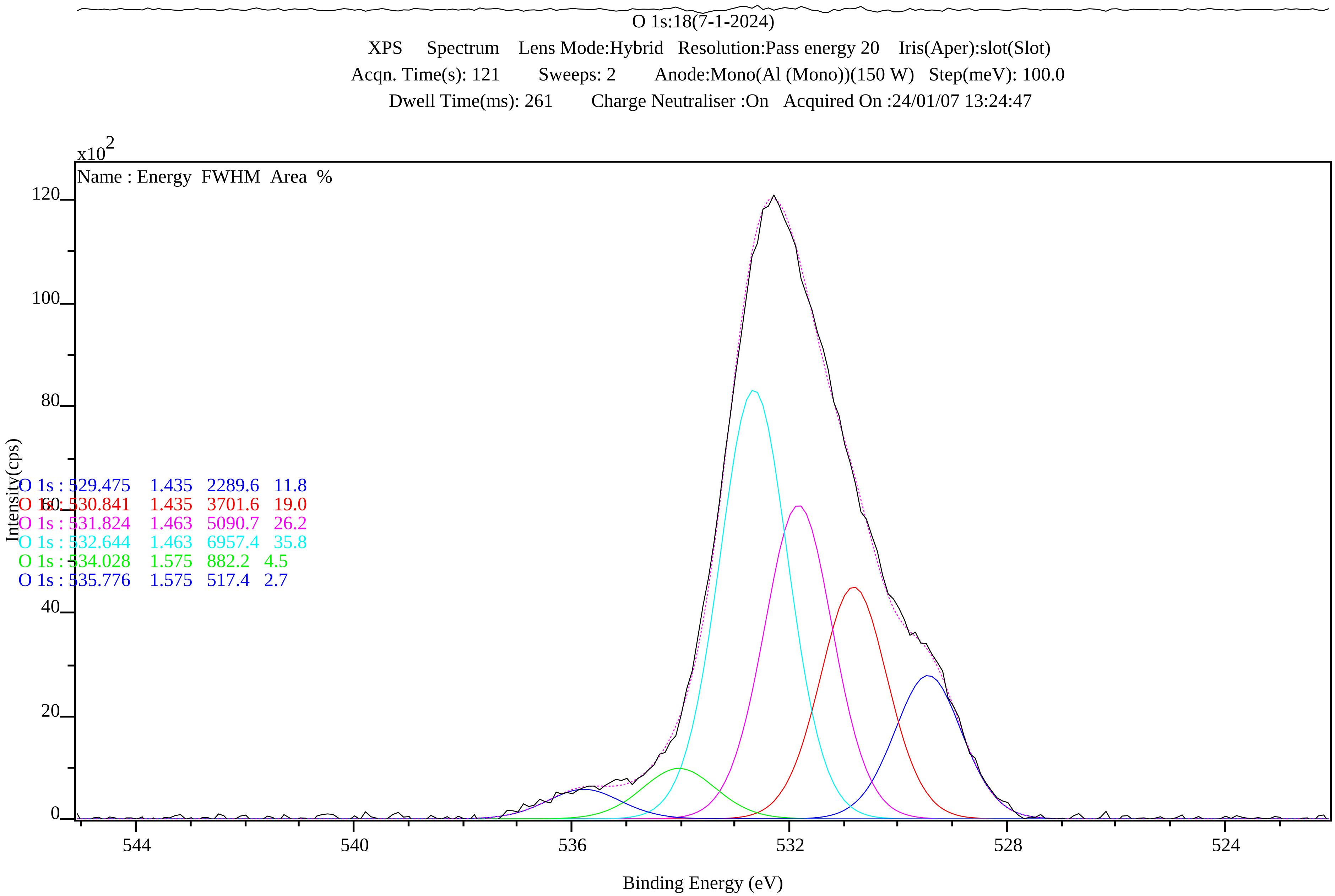
O 1s:18(7-1-2024)
XPS Spectrum Lens Mode:Hybrid Resolution:Pass energy 20 Iris(Aper):slot(Slot)
Acqn. Time(s): 121 Sweeps: 2 Anode:Mono(Al (Mono))(150 W) Step(meV): 100.0
Dwell Time(ms): 261 Charge Neutraliser :On Acquired On :24/01/07 13:24:47
x10^2
Name : Energy FWHM Area %
O 1s : 529.475 1.435 2289.6 11.8
O 1s : 530.841 1.435 3701.6 19.0
O 1s : 531.824 1.463 5090.7 26.2
O 1s : 532.644 1.463 6957.4 35.8
O 1s : 534.028 1.575 882.2 4.5
O 1s : 535.776 1.575 517.4 2.7
Intensity(cps)
0
20
40
60
80
100
120
544
540
536
532
528
524
Binding Energy (eV)

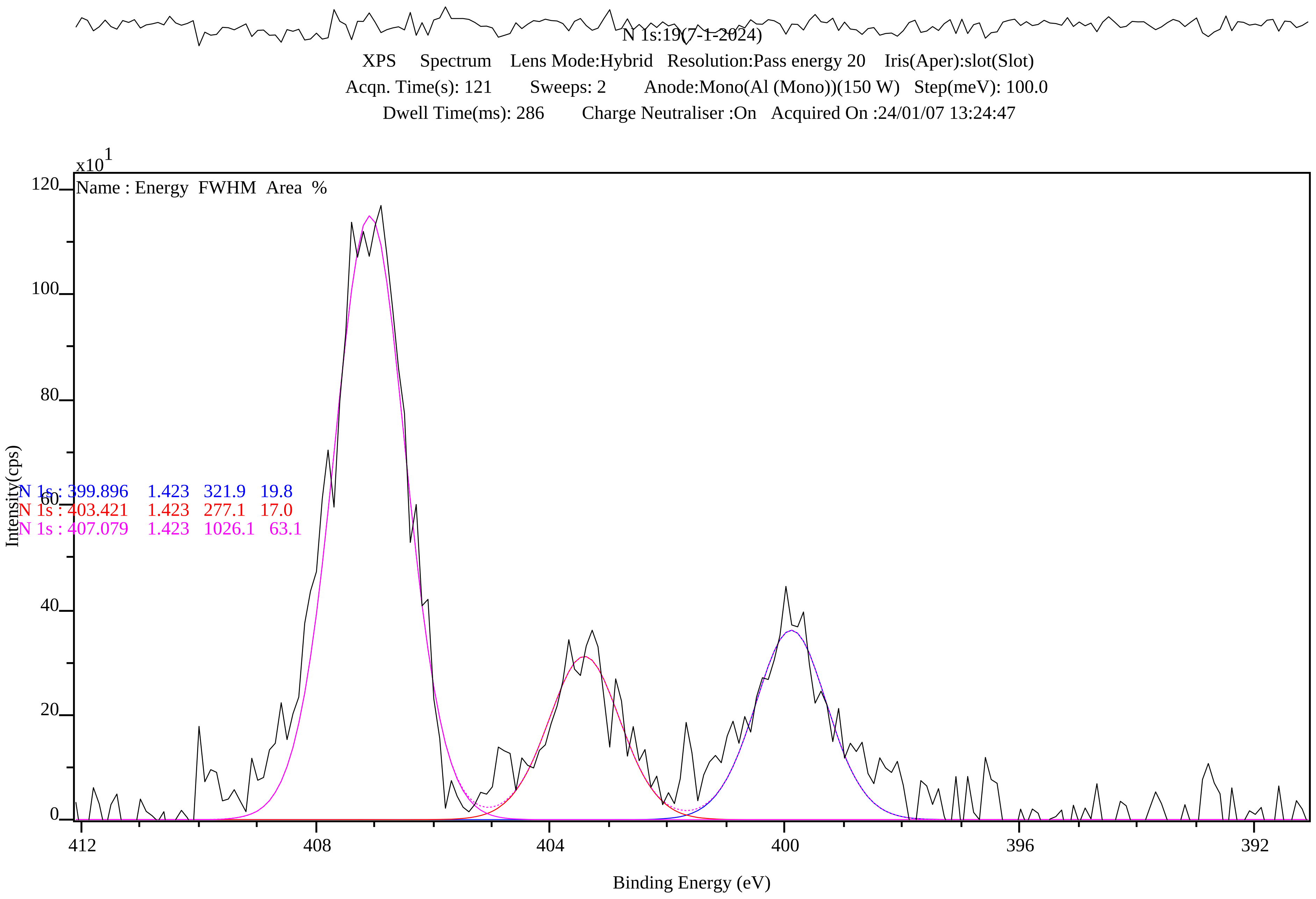
N 1s:19(7-1-2024)
XPS Spectrum Lens Mode:Hybrid Resolution:Pass energy 20 Iris(Aper):slot(Slot)
Acqn. Time(s): 121 Sweeps: 2 Anode:Mono(Al (Mono))(150 W) Step(meV): 100.0
Dwell Time(ms): 286 Charge Neutraliser :On Acquired On :24/01/07 13:24:47
x10^1
Name : Energy FWHM Area %
N 1s : 399.896 1.423 321.9 19.8
N 1s : 403.421 1.423 277.1 17.0
N 1s : 407.079 1.423 1026.1 63.1
Intensity(cps)
120
100
80
60
40
20
0
412
408
404
400
396
392
Binding Energy (eV)

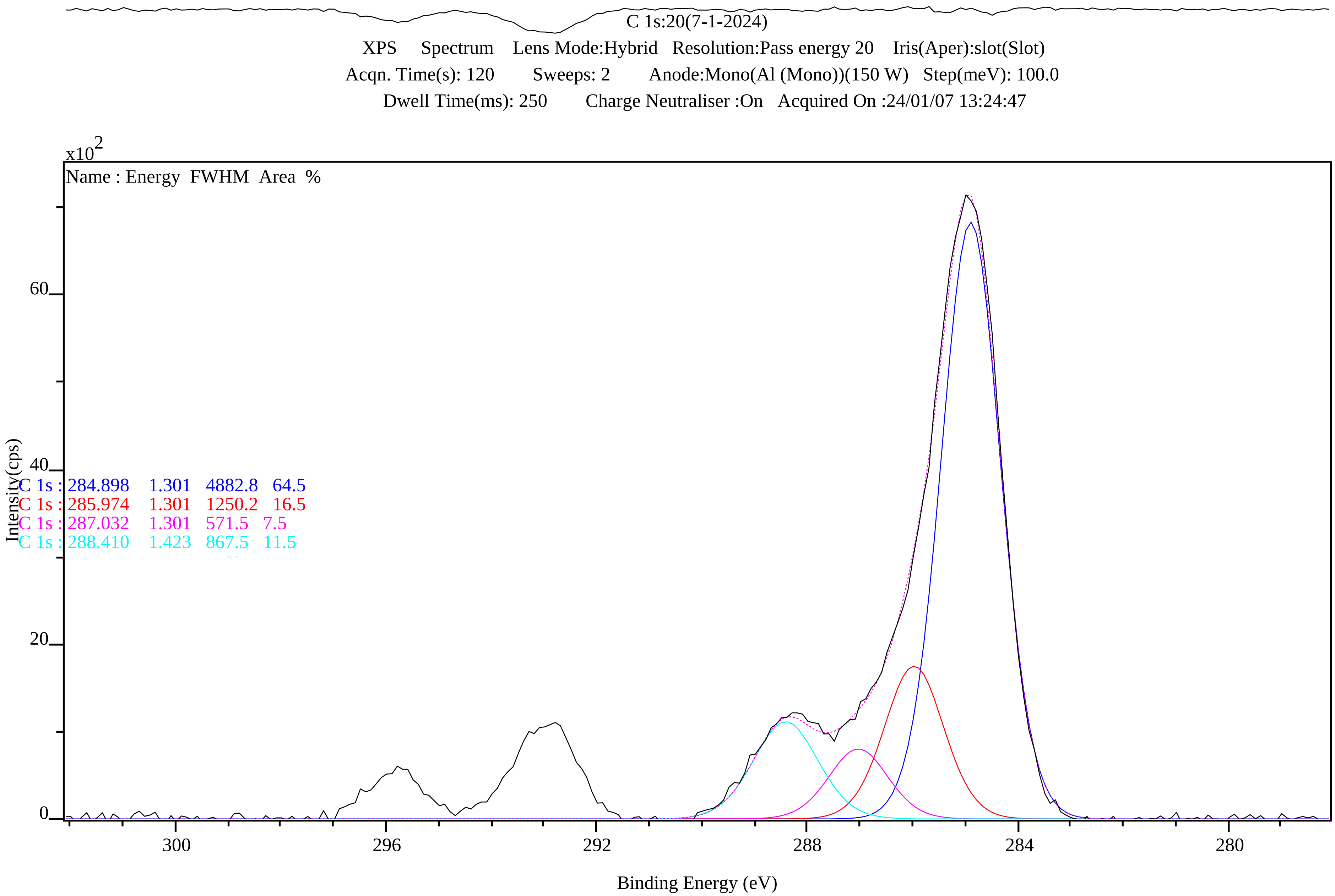
C 1s:20(7-1-2024)
XPS Spectrum Lens Mode:Hybrid Resolution:Pass energy 20 Iris(Aper):slot(Slot)
Acqn. Time(s): 120 Sweeps: 2 Anode:Mono(Al (Mono))(150 W) Step(meV): 100.0
Dwell Time(ms): 250 Charge Neutraliser :On Acquired On :24/01/07 13:24:47
x10^2
Name : Energy FWHM Area %
C 1s : 284.898 1.301 4882.8 64.5
C 1s : 285.974 1.301 1250.2 16.5
C 1s : 287.032 1.301 571.5 7.5
C 1s : 288.410 1.423 867.5 11.5
Intensity(cps)
60
40
20
0
300
296
292
288
284
280
Binding Energy (eV)
