# Supplementary material for: Enhancing the Longevity and Structural Stability of Humidity Sensors: Iron Thin Films with Nitride Bonding Synthesized via Magnetic Field-Assisted Sparking Discharge
Source: Sensors (Basel). 2024 Aug 23;24(17):5466. doi: 10.3390/s24175466 (PMC11397896; doi:10.3390/s24175466)
Supplement: Supplementary file 1 [file sensors-24-05466-s001.zip › XPS_7-1-2024/3.IRON N2 mag sample2/report.pdf]

Quantification Report

/C=/data/7-1-2024/7-1-2024.dset Fri Jan 12 13:26:11 2024

State : Angle Name : Position 3

Peak	Type	Position BE (eV)	FWHM (eV)	Raw Area (cps eV)	RSF	Atomic Mass	Atomic Conc %	Mass Conc %
Na 1s	Reg	1071.000	2.309	68965.0	1.685	22.990	4.71	6.22
Fe 2p	Reg	711.000	4.435	127890.0	2.957	55.846	5.36	17.21
O 1s	Reg	532.000	3.819	223600.0	0.780	15.999	36.74	33.77
N 1s	Reg	407.000	2.591	18820.0	0.477	14.007	5.20	4.19
Ca 2p	Reg	348.000	2.117	4180.0	1.833	40.078	0.31	0.71
K 2p	Reg	293.000	3.829	15245.0	1.466	39.102	1.44	3.23
C 1s	Reg	285.000	2.799	87315.0	0.278	12.011	43.23	29.83
Si 2p	Reg	104.000	2.833	7430.0	0.328	28.086	3.01	4.85
