# Supplementary material for: Enhancing the Longevity and Structural Stability of Humidity Sensors: Iron Thin Films with Nitride Bonding Synthesized via Magnetic Field-Assisted Sparking Discharge
Source: Sensors (Basel). 2024 Aug 23;24(17):5466. doi: 10.3390/s24175466 (PMC11397896; doi:10.3390/s24175466)
Supplement: Supplementary file 1 [file sensors-24-05466-s001.zip › XPS_7-1-2024/4.IRON O2 magnet/component.pdf]

Fe 2p:24(7-1-2024)

XPS Spectrum Lens Mode:Hybrid Resolution:Pass energy 20 Iris(Aper):slot(Slot)

Acqn. Time(s): 120 Sweeps: 2 Anode:Mono(Al (Mono))(150 W) Step(meV): 100.0

Dwell Time(ms): 150 Charge Neutraliser :On Acquired On :24/01/07 13:37:57

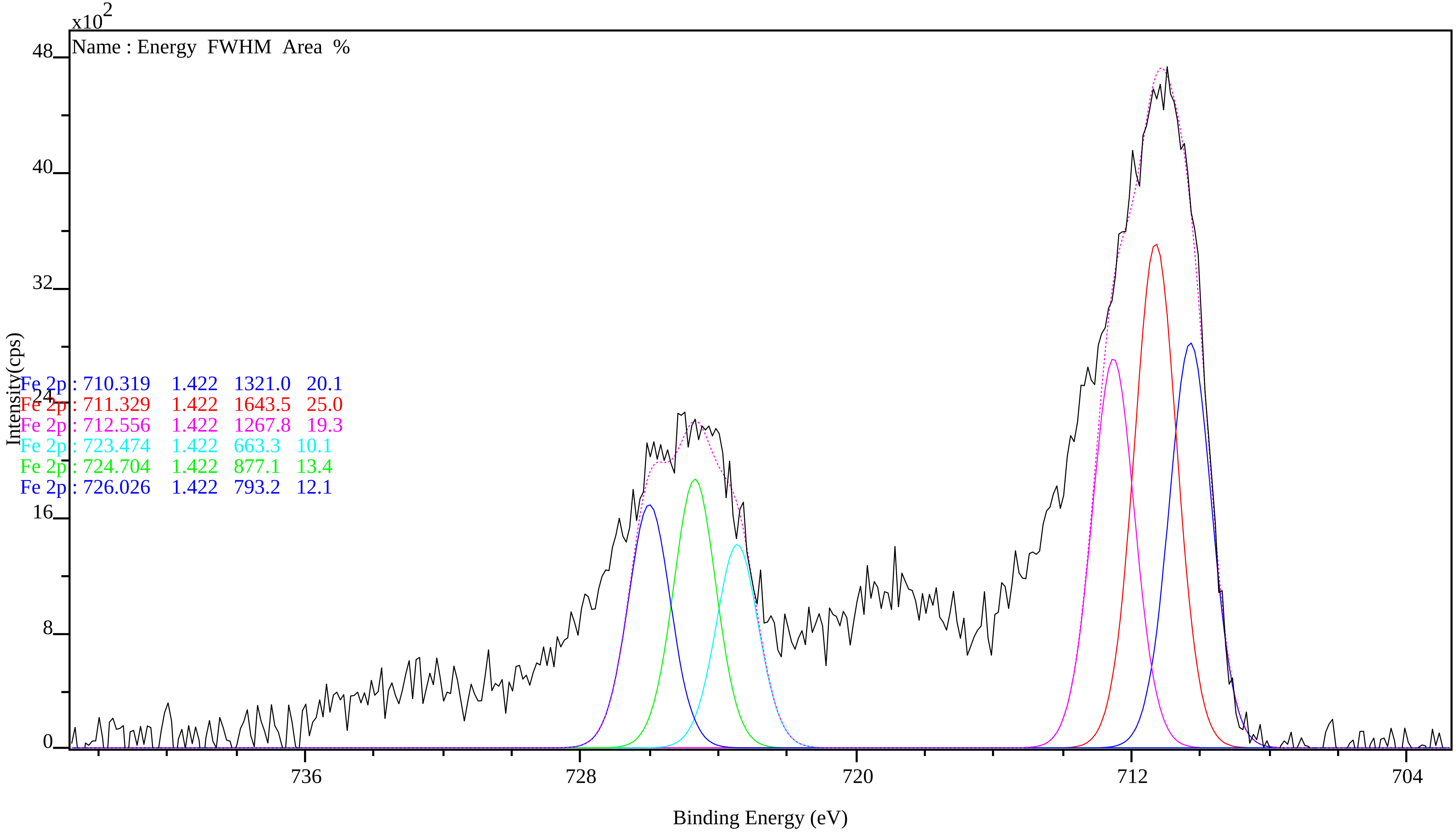

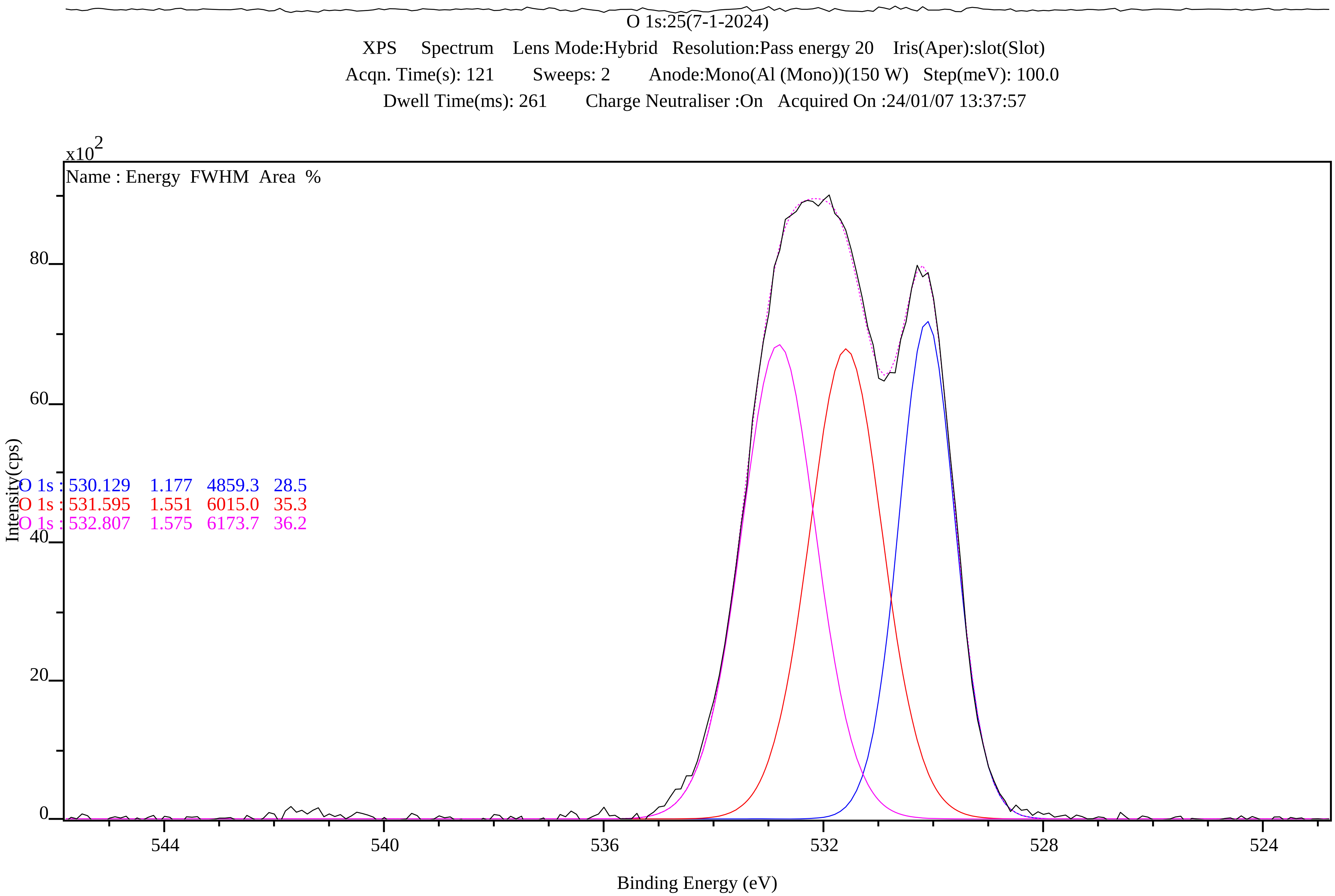
O 1s:25(7-1-2024)
XPS Spectrum Lens Mode:Hybrid Resolution:Pass energy 20 Iris(Aper):slot(Slot)
Acqn. Time(s): 121 Sweeps: 2 Anode:Mono(Al (Mono))(150 W) Step(meV): 100.0
Dwell Time(ms): 261 Charge Neutraliser :On Acquired On :24/01/07 13:37:57
x10^2
Name : Energy FWHM Area %
O 1s : 530.129 1.177 4859.3 28.5
O 1s : 531.595 1.551 6015.0 35.3
O 1s : 532.807 1.575 6173.7 36.2
Intensity(cps)
80
60
40
20
0
544
540
536
532
528
524
Binding Energy (eV)

N 1s:26(7-1-2024)

XPS Spectrum Lens Mode:Hybrid Resolution:Pass energy 20 Iris(Aper):slot(Slot)

Acqn. Time(s): 121 Sweeps: 2 Anode:Mono(Al (Mono))(150 W) Step(meV): 100.0

Dwell Time(ms): 286 Charge Neutraliser :On Acquired On :24/01/07 13:37:57

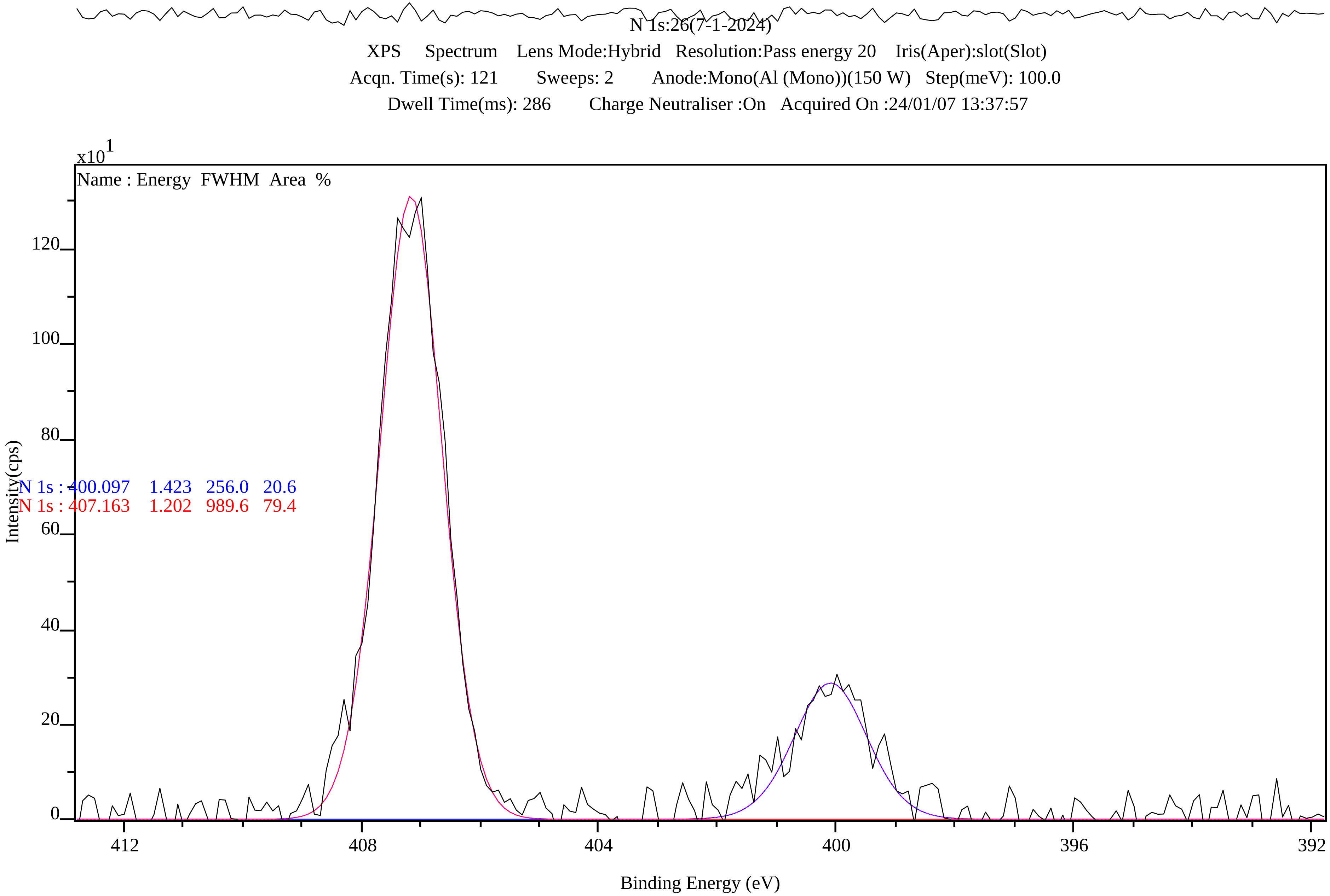

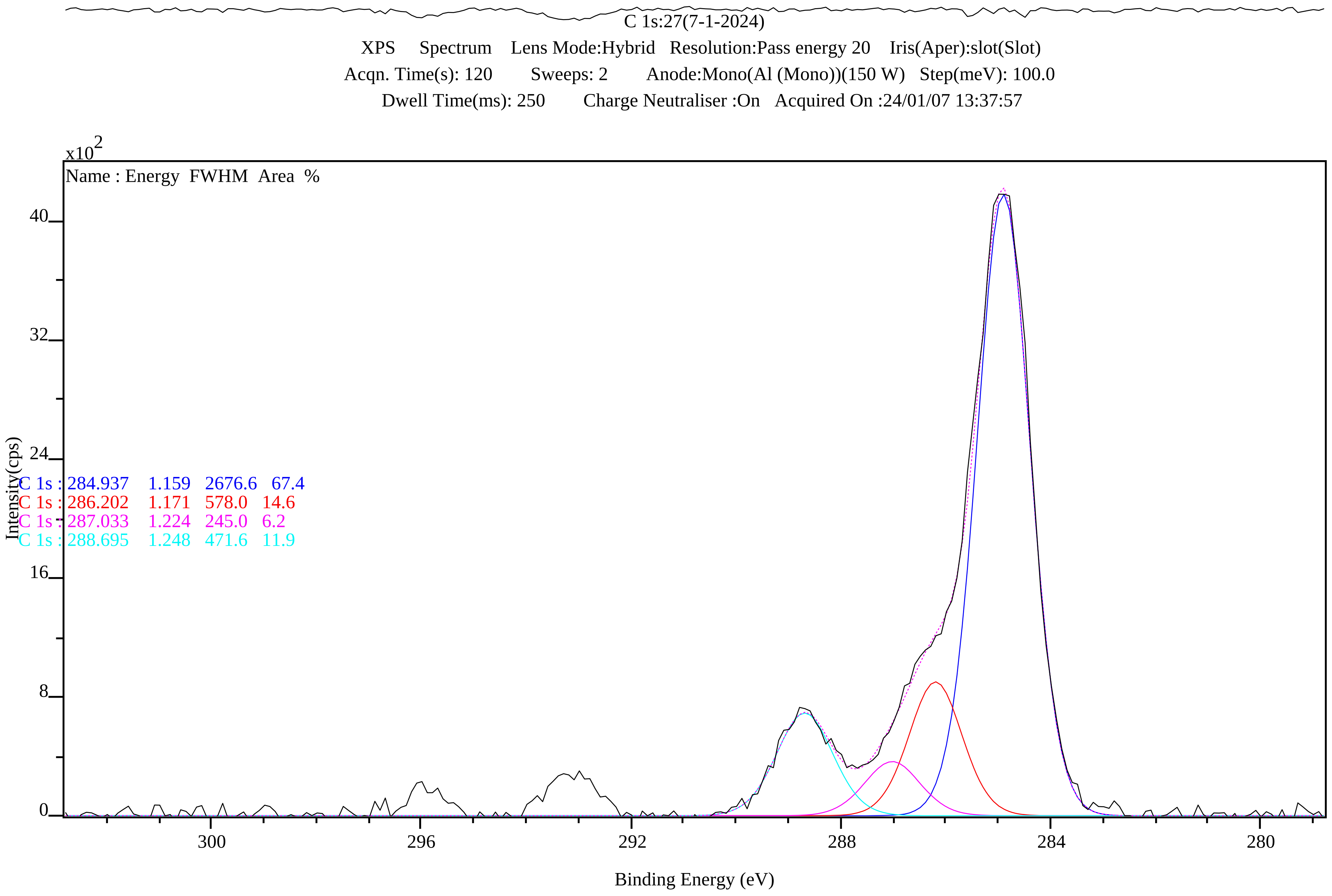
C 1s:27(7-1-2024)
XPS Spectrum Lens Mode:Hybrid Resolution:Pass energy 20 Iris(Aper):slot(Slot)
Acqn. Time(s): 120 Sweeps: 2 Anode:Mono(Al (Mono))(150 W) Step(meV): 100.0
Dwell Time(ms): 250 Charge Neutraliser :On Acquired On :24/01/07 13:37:57
x10^2
Name : Energy FWHM Area %
C 1s : 284.937 1.159 2676.6 67.4
C 1s : 286.202 1.171 578.0 14.6
C 1s : 287.033 1.224 245.0 6.2
C 1s : 288.695 1.248 471.6 11.9
Intensity(cps)
40
32
24
16
8
0
300
296
292
288
284
280
Binding Energy (eV)
