# Supplementary material for: Enhancing the Longevity and Structural Stability of Humidity Sensors: Iron Thin Films with Nitride Bonding Synthesized via Magnetic Field-Assisted Sparking Discharge
Source: Sensors (Basel). 2024 Aug 23;24(17):5466. doi: 10.3390/s24175466 (PMC11397896; doi:10.3390/s24175466)
Supplement: Supplementary file 1 [file sensors-24-05466-s001.zip › XPS_7-1-2024/4.IRON O2 magnet/report.pdf]

Quantification Report

/C=/data/7-1-2024/7-1-2024.dset Fri Jan 12 13:27:00 2024

State : Angle Name : Position 4

Peak	Type	Position BE (eV)	FWHM (eV)	Raw Area (cps eV)	RSF	Atomic Mass	Atomic Conc %	Mass Conc %
Na 1s	Reg	1072.000	2.052	10260.0	1.685	22.990	0.84	0.86
Zn 2p	Reg	1022.000	2.106	41355.0	5.589	65.387	1.04	3.03
Fe 2p	Reg	711.000	4.943	329420.0	2.957	55.846	16.63	41.41
O 1s	Reg	532.000	4.026	223605.0	0.780	15.999	44.21	31.55
N 1s	Reg	407.000	2.046	12560.0	0.477	14.007	4.18	2.61
K 2p	Reg	293.000	1.629	2220.0	1.466	39.102	0.25	0.44
C 1s	Reg	285.000	2.362	49300.0	0.278	12.011	29.37	15.73
Si 2p	Reg	103.000	4.161	7140.0	0.328	28.086	3.47	4.35
