# Supplementary material for: Enhancing the Longevity and Structural Stability of Humidity Sensors: Iron Thin Films with Nitride Bonding Synthesized via Magnetic Field-Assisted Sparking Discharge
Source: Sensors (Basel). 2024 Aug 23;24(17):5466. doi: 10.3390/s24175466 (PMC11397896; doi:10.3390/s24175466)
Supplement: Supplementary file 1 [file sensors-24-05466-s001.zip › XPS_7-1-2024/5.IRON N2 sample2/component.pdf]

Fe 2p:31(7-1-2024)

XPS Spectrum Lens Mode:Hybrid Resolution:Pass energy 20 Iris(Aper):slot(Slot)

Acqn. Time(s): 120 Sweeps: 2 Anode:Mono(Al (Mono))(150 W) Step(meV): 100.0

Dwell Time(ms): 150 Charge Neutraliser :On Acquired On :24/01/07 13:51:21

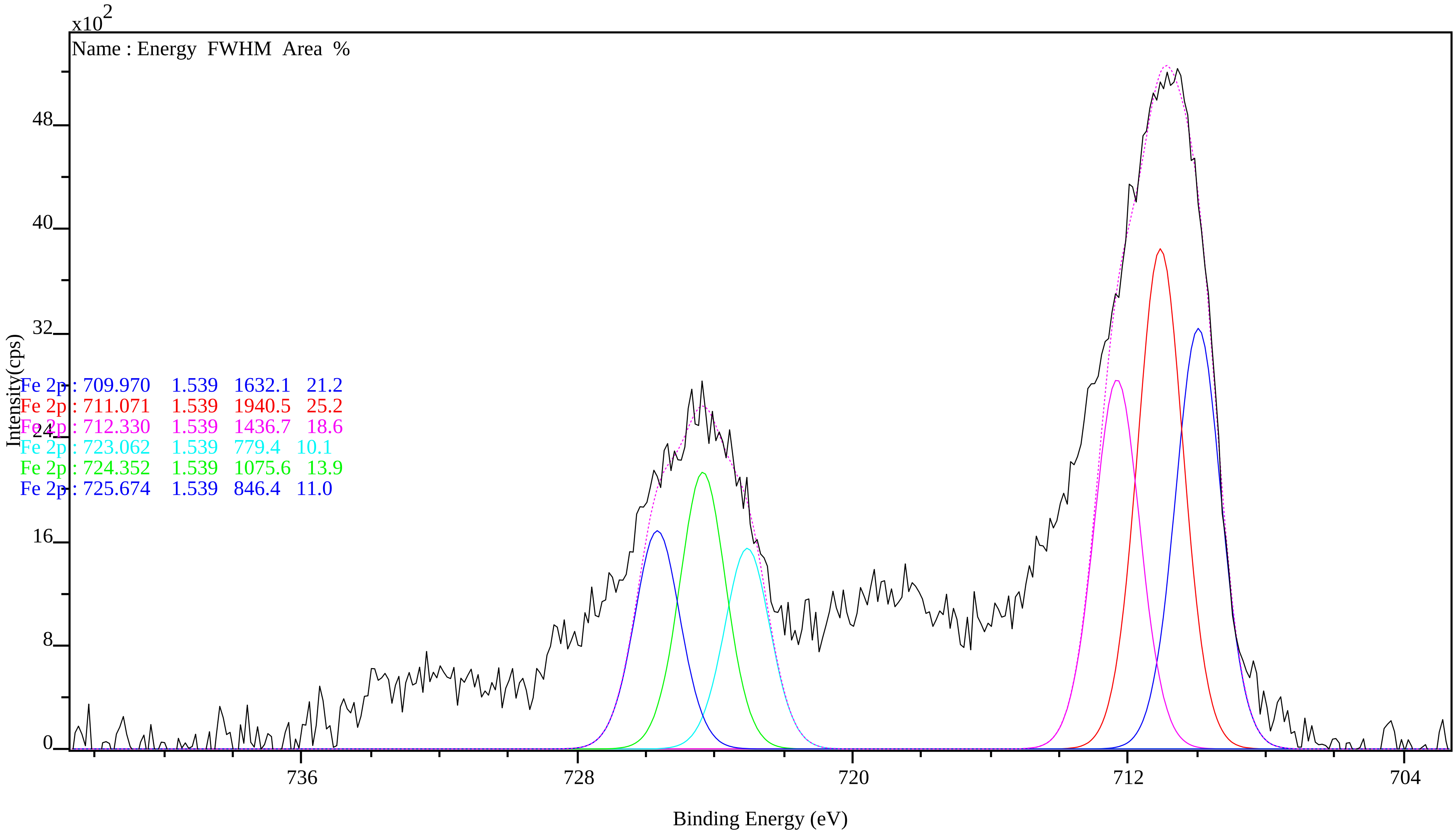

O 1s:32(7-1-2024)

XPS Spectrum Lens Mode:Hybrid Resolution:Pass energy 20 Iris(Aper):slot(Slot)

Acqn. Time(s): 121 Sweeps: 2 Anode:Mono(Al (Mono))(150 W) Step(meV): 100.0

Dwell Time(ms): 261 Charge Neutraliser :On Acquired On :24/01/07 13:51:21

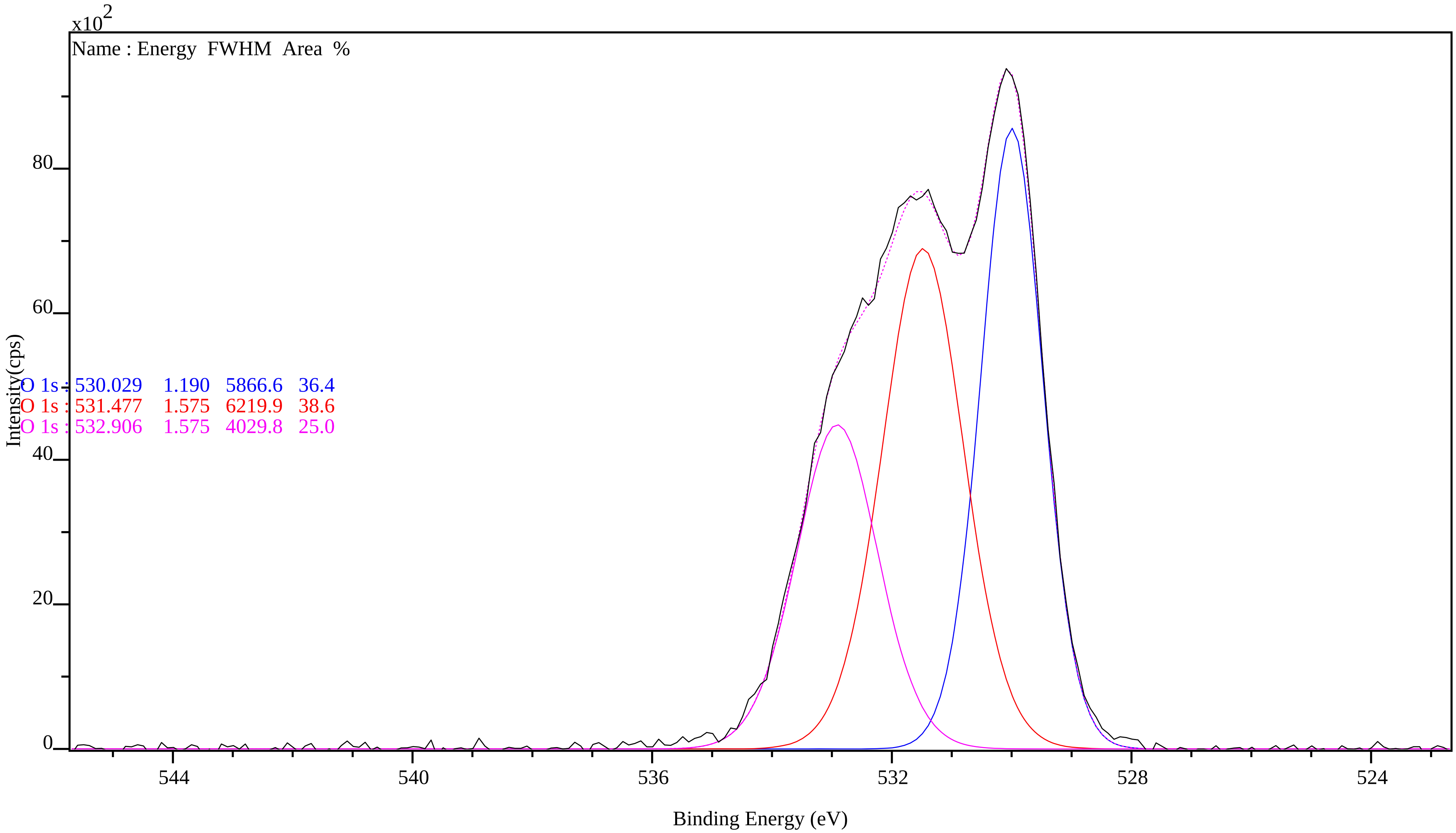

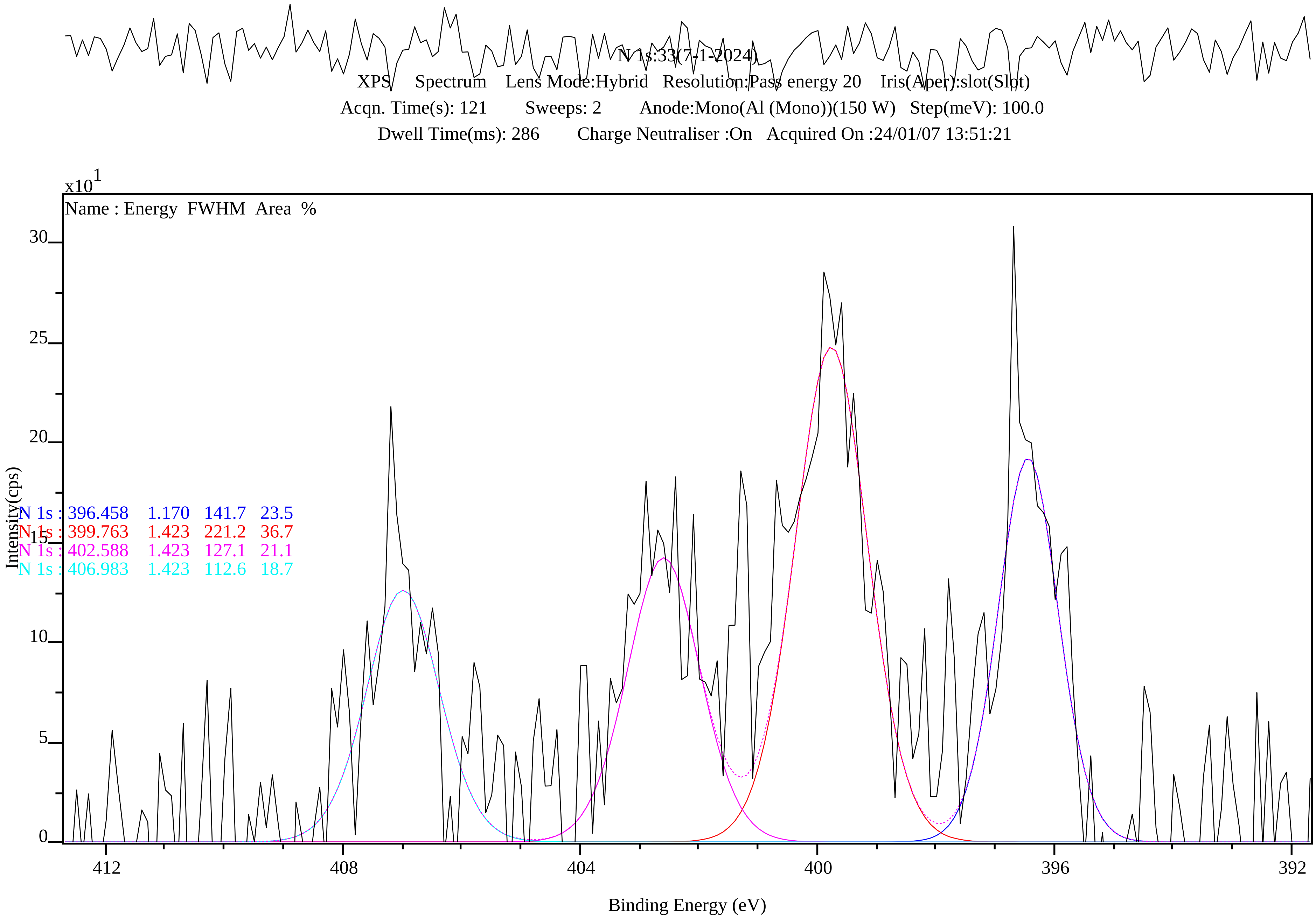
N 1s:33(7-1-2024)
XPS Spectrum Lens Mode:Hybrid Resolution:Pass energy 20 Iris(Aper):slot(Slot)
Acqn. Time(s): 121 Sweeps: 2 Anode:Mono(Al (Mono))(150 W) Step(meV): 100.0
Dwell Time(ms): 286 Charge Neutraliser :On Acquired On :24/01/07 13:51:21
x10^1
Name : Energy FWHM Area %
N 1s : 396.458 1.170 141.7 23.5
N 1s : 399.763 1.423 221.2 36.7
N 1s : 402.588 1.423 127.1 21.1
N 1s : 406.983 1.423 112.6 18.7
Intensity(cps)
30
25
20
15
10
5
0
412
408
404
400
396
392
Binding Energy (eV)

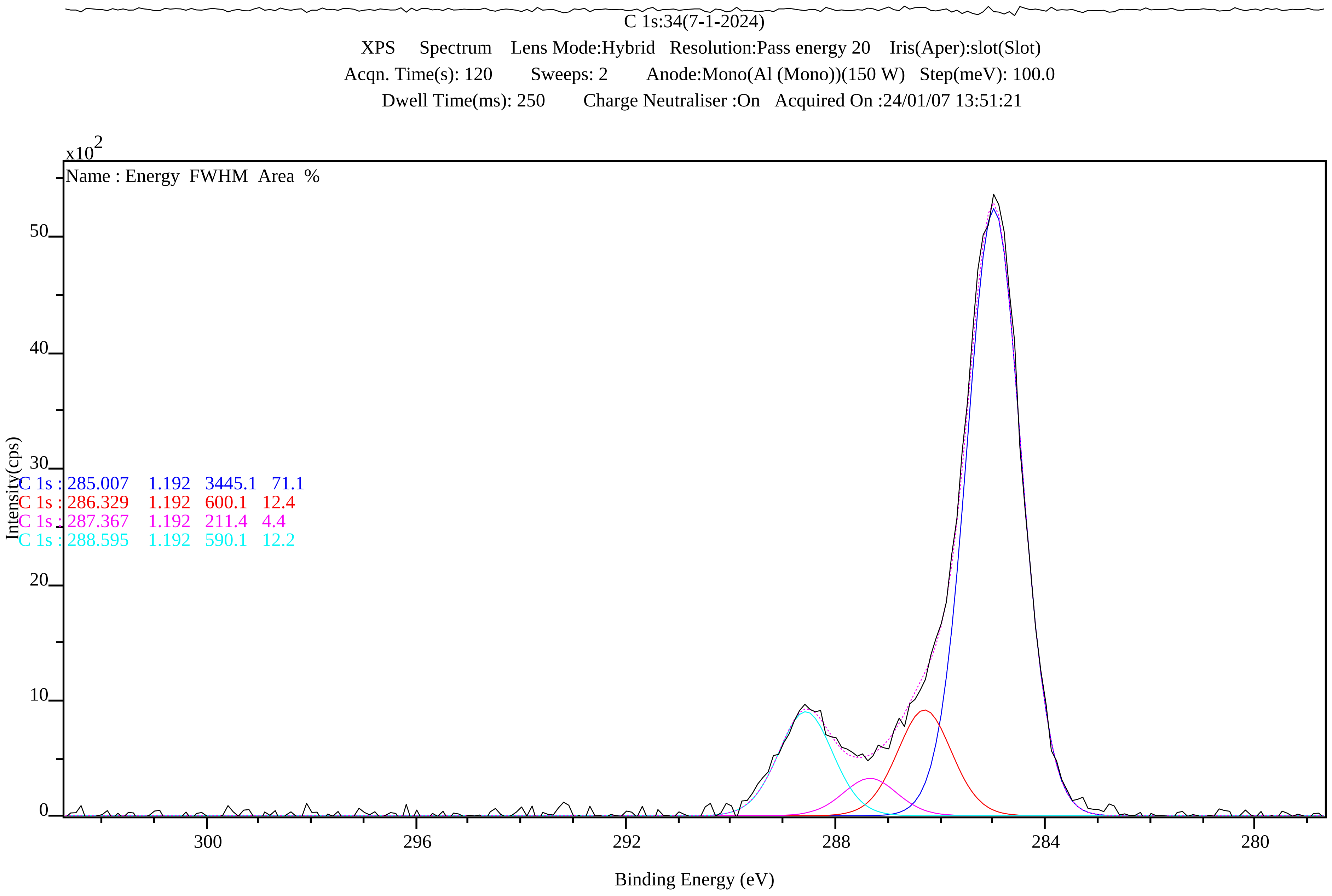
C 1s:34(7-1-2024)
XPS Spectrum Lens Mode:Hybrid Resolution:Pass energy 20 Iris(Aper):slot(Slot)
Acqn. Time(s): 120 Sweeps: 2 Anode:Mono(Al (Mono))(150 W) Step(meV): 100.0
Dwell Time(ms): 250 Charge Neutraliser :On Acquired On :24/01/07 13:51:21
x10^2
Name : Energy FWHM Area %
C 1s : 285.007 1.192 3445.1 71.1
C 1s : 286.329 1.192 600.1 12.4
C 1s : 287.367 1.192 211.4 4.4
C 1s : 288.595 1.192 590.1 12.2
Intensity(cps)
0
10
20
30
40
50
300
296
292
288
284
280
Binding Energy (eV)
