# Supplementary material for: Enhancing the Longevity and Structural Stability of Humidity Sensors: Iron Thin Films with Nitride Bonding Synthesized via Magnetic Field-Assisted Sparking Discharge
Source: Sensors (Basel). 2024 Aug 23;24(17):5466. doi: 10.3390/s24175466 (PMC11397896; doi:10.3390/s24175466)
Supplement: Supplementary file 1 [file sensors-24-05466-s001.zip › XPS_7-1-2024/5.IRON N2 sample2/report.pdf]

Quantification Report

/C=/data/7-1-2024/7-1-2024.dset Fri Jan 12 13:28:16 2024

State : Angle Name : Position 5

Peak	Type	Position BE (eV)	FWHM (eV)	Raw Area (cps eV)	RSF	Atomic Mass	Atomic Conc %	Mass Conc %
Zn 2p	Reg	1022.000	3.721	10670.0	5.589	65.387	0.26	0.75
Fe 2p	Reg	711.000	4.981	386745.0	2.957	55.846	18.80	46.83
O 1s	Reg	531.000	4.231	208730.0	0.780	15.999	39.75	28.38
N 1s	Reg	399.000	5.084	5150.0	0.477	14.007	1.65	1.03
C 1s	Reg	285.000	2.219	64520.0	0.278	12.011	37.01	19.83
Si 2p	Reg	104.000	2.631	5400.0	0.328	28.086	2.53	3.17
