# Supplementary material for: Enhancing the Longevity and Structural Stability of Humidity Sensors: Iron Thin Films with Nitride Bonding Synthesized via Magnetic Field-Assisted Sparking Discharge
Source: Sensors (Basel). 2024 Aug 23;24(17):5466. doi: 10.3390/s24175466 (PMC11397896; doi:10.3390/s24175466)
Supplement: Supplementary file 1 [file sensors-24-05466-s001.zip › XPS_7-1-2024/6.IRON O2 no magnet/component.pdf]

Fe 2p:38(7-1-2024)

XPS Spectrum Lens Mode:Hybrid Resolution:Pass energy 20 Iris(Aper):slot(Slot)

Acqn. Time(s): 120 Sweeps: 2 Anode:Mono(Al (Mono))(150 W) Step(meV): 100.0

Dwell Time(ms): 150 Charge Neutraliser :On Acquired On :24/01/07 14:04:29

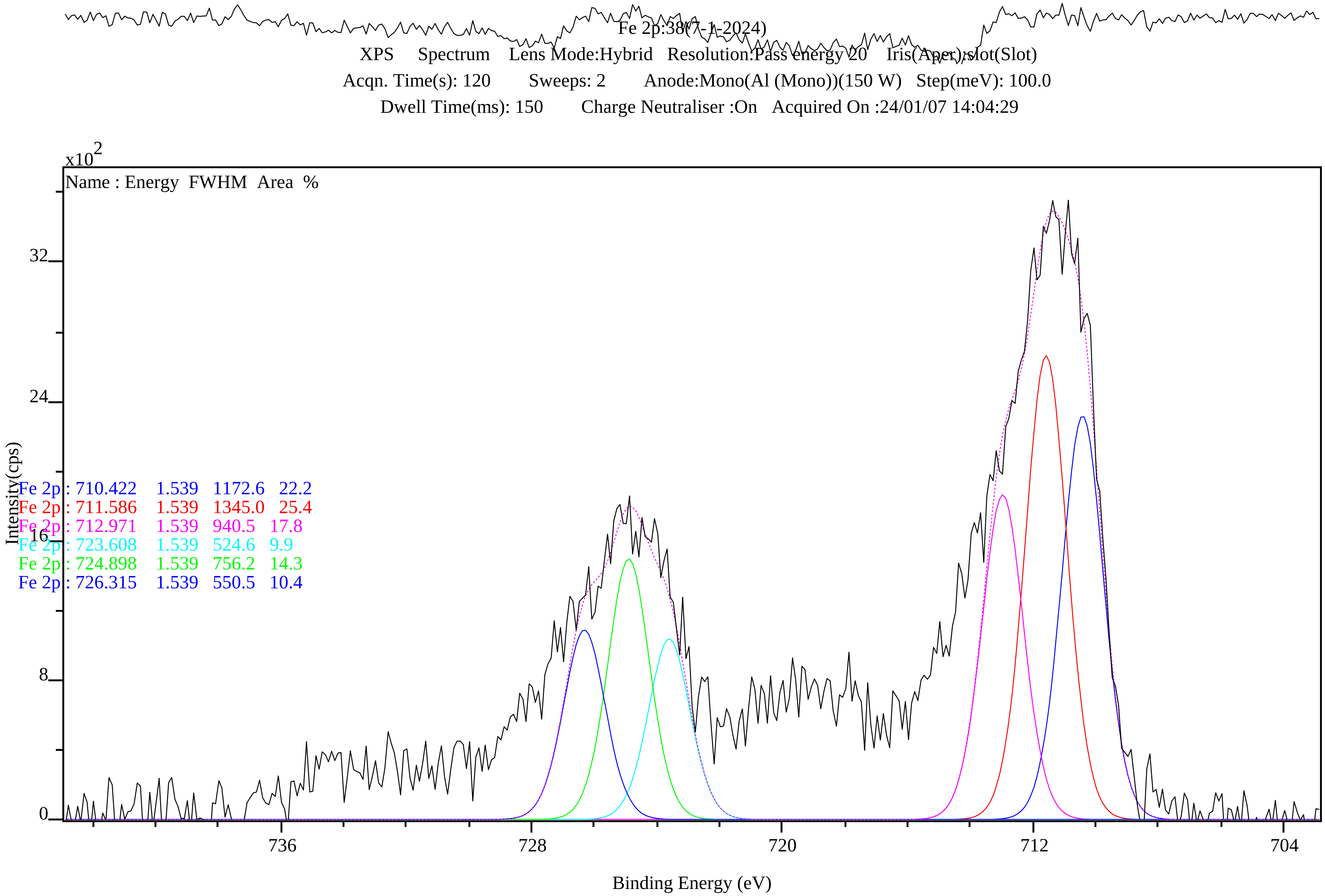

O 1s:39(7-1-2024)

XPS Spectrum Lens Mode:Hybrid Resolution:Pass energy 20 Iris(Aper):slot(Slot)

Acqn. Time(s): 121 Sweeps: 2 Anode:Mono(Al (Mono))(150 W) Step(meV): 100.0

Dwell Time(ms): 261 Charge Neutraliser :On Acquired On :24/01/07 14:04:29

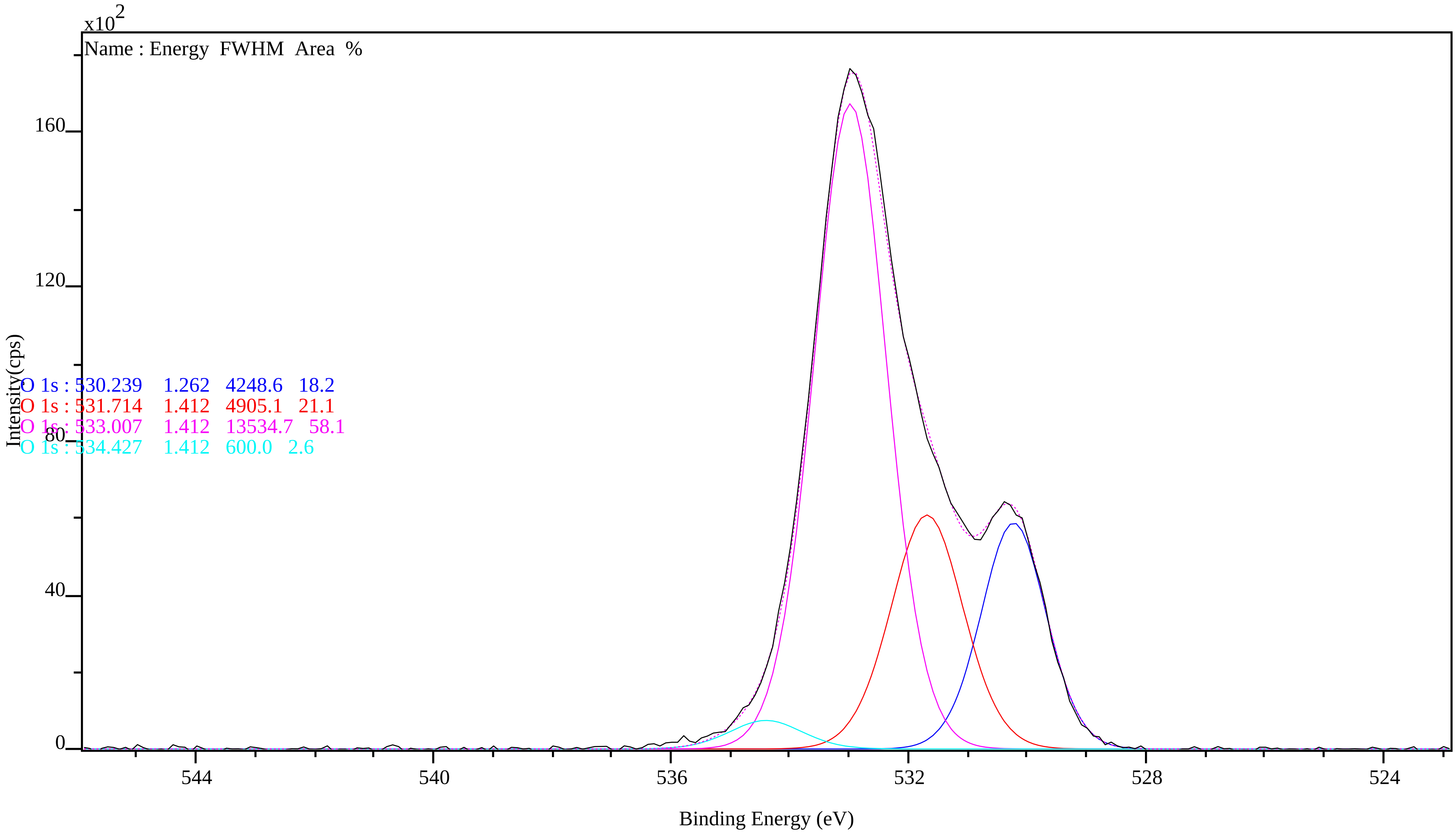

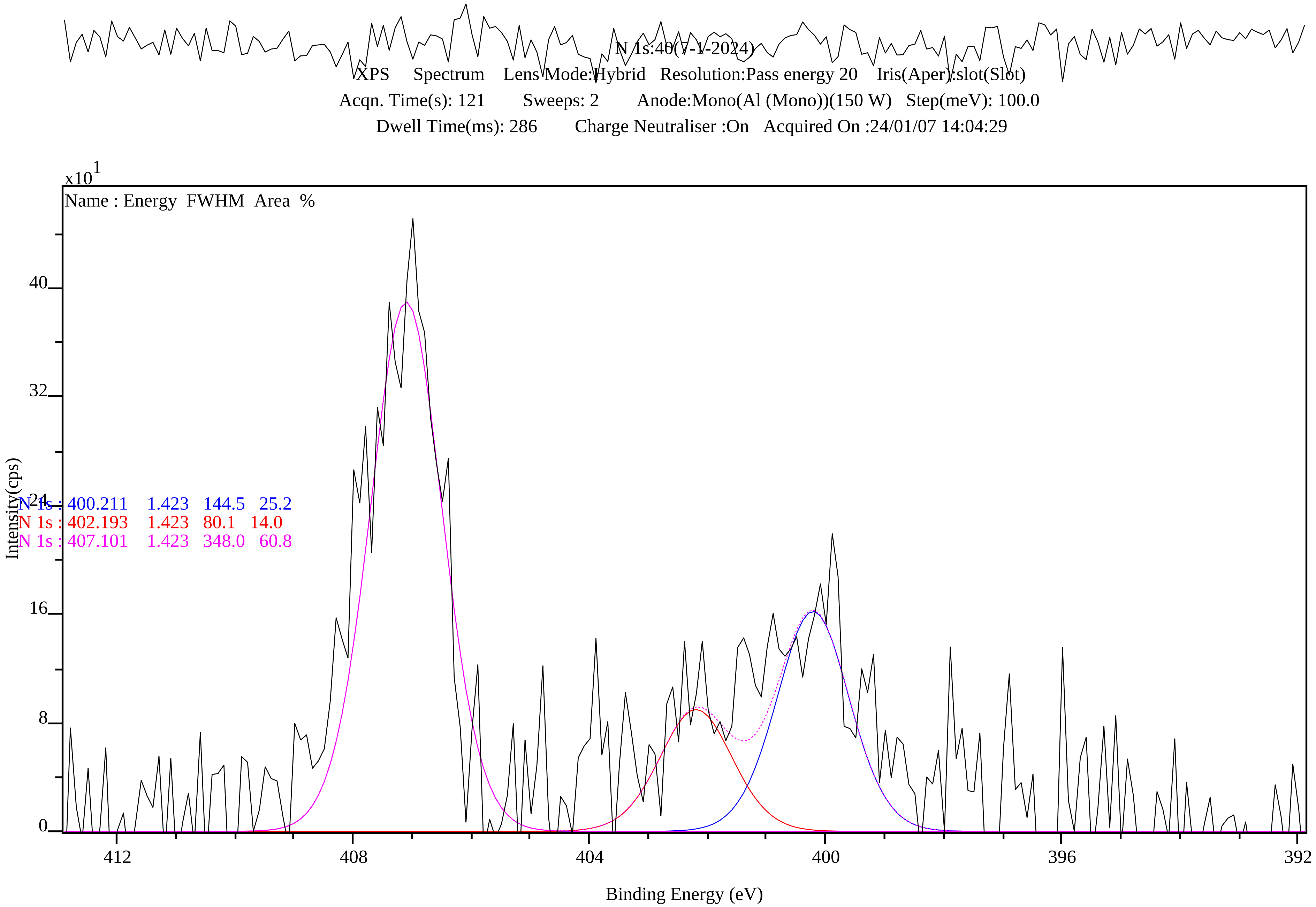
N 1s:40(7-1-2024)
XPS Spectrum Lens Mode:Hybrid Resolution:Pass energy 20 Iris(Aper):slot(Slot)
Acqn. Time(s): 121 Sweeps: 2 Anode:Mono(Al (Mono))(150 W) Step(meV): 100.0
Dwell Time(ms): 286 Charge Neutraliser :On Acquired On :24/01/07 14:04:29
x10^1
Name : Energy FWHM Area %
N 1s : 400.211 1.423 144.5 25.2
N 1s : 402.193 1.423 80.1 14.0
N 1s : 407.101 1.423 348.0 60.8
Intensity(cps)
0
8
16
24
32
40
412
408
404
400
396
392
Binding Energy (eV)

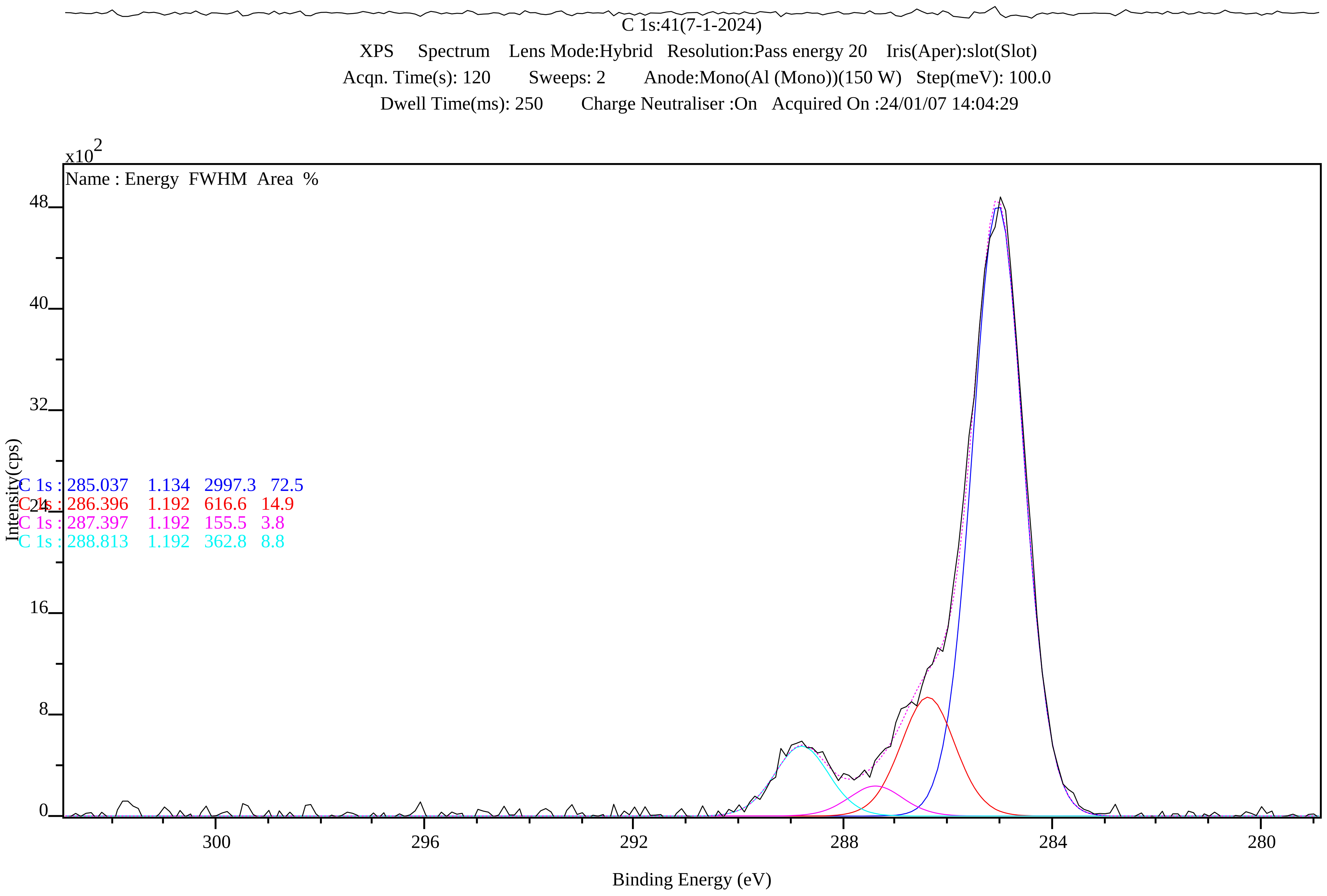
C 1s:41(7-1-2024)
XPS Spectrum Lens Mode:Hybrid Resolution:Pass energy 20 Iris(Aper):slot(Slot)
Acqn. Time(s): 120 Sweeps: 2 Anode:Mono(Al (Mono))(150 W) Step(meV): 100.0
Dwell Time(ms): 250 Charge Neutraliser :On Acquired On :24/01/07 14:04:29
x10^2
Name : Energy FWHM Area %
C 1s : 285.037 1.134 2997.3 72.5
C 1s : 286.396 1.192 616.6 14.9
C 1s : 287.397 1.192 155.5 3.8
C 1s : 288.813 1.192 362.8 8.8
Intensity(cps)
48
40
32
24
16
8
0
300
296
292
288
284
280
Binding Energy (eV)
