# Supplementary material for: Enhancing the Longevity and Structural Stability of Humidity Sensors: Iron Thin Films with Nitride Bonding Synthesized via Magnetic Field-Assisted Sparking Discharge
Source: Sensors (Basel). 2024 Aug 23;24(17):5466. doi: 10.3390/s24175466 (PMC11397896; doi:10.3390/s24175466)
Supplement: Supplementary file 1 [file sensors-24-05466-s001.zip › XPS_7-1-2024/6.IRON O2 no magnet/report.pdf]

Quantification Report

/C=/data/7-1-2024/7-1-2024.dset Fri Jan 12 13:29:34 2024

State : Angle Name : Position 6

Peak	Type	Position BE (eV)	FWHM (eV)	Raw Area (cps eV)	RSF	Atomic Mass	Atomic Conc %	Mass Conc %
Zn 2p	Reg	1022.000	2.501	19485.0	5.589	65.387	0.42	1.33
Fe 2p	Reg	711.000	4.873	247930.0	2.957	55.846	10.77	29.05
O 1s	Reg	532.000	3.310	284490.0	0.780	15.999	48.42	37.41
N 1s	Reg	407.000	2.208	5300.0	0.477	14.007	1.52	1.03
C 1s	Reg	285.000	2.251	54140.0	0.278	12.011	27.76	16.10
Si 2p	Reg	103.000	2.344	26530.0	0.328	28.086	11.11	15.07
