# Supplementary material for: Enhancing the Longevity and Structural Stability of Humidity Sensors: Iron Thin Films with Nitride Bonding Synthesized via Magnetic Field-Assisted Sparking Discharge
Source: Sensors (Basel). 2024 Aug 23;24(17):5466. doi: 10.3390/s24175466 (PMC11397896; doi:10.3390/s24175466)

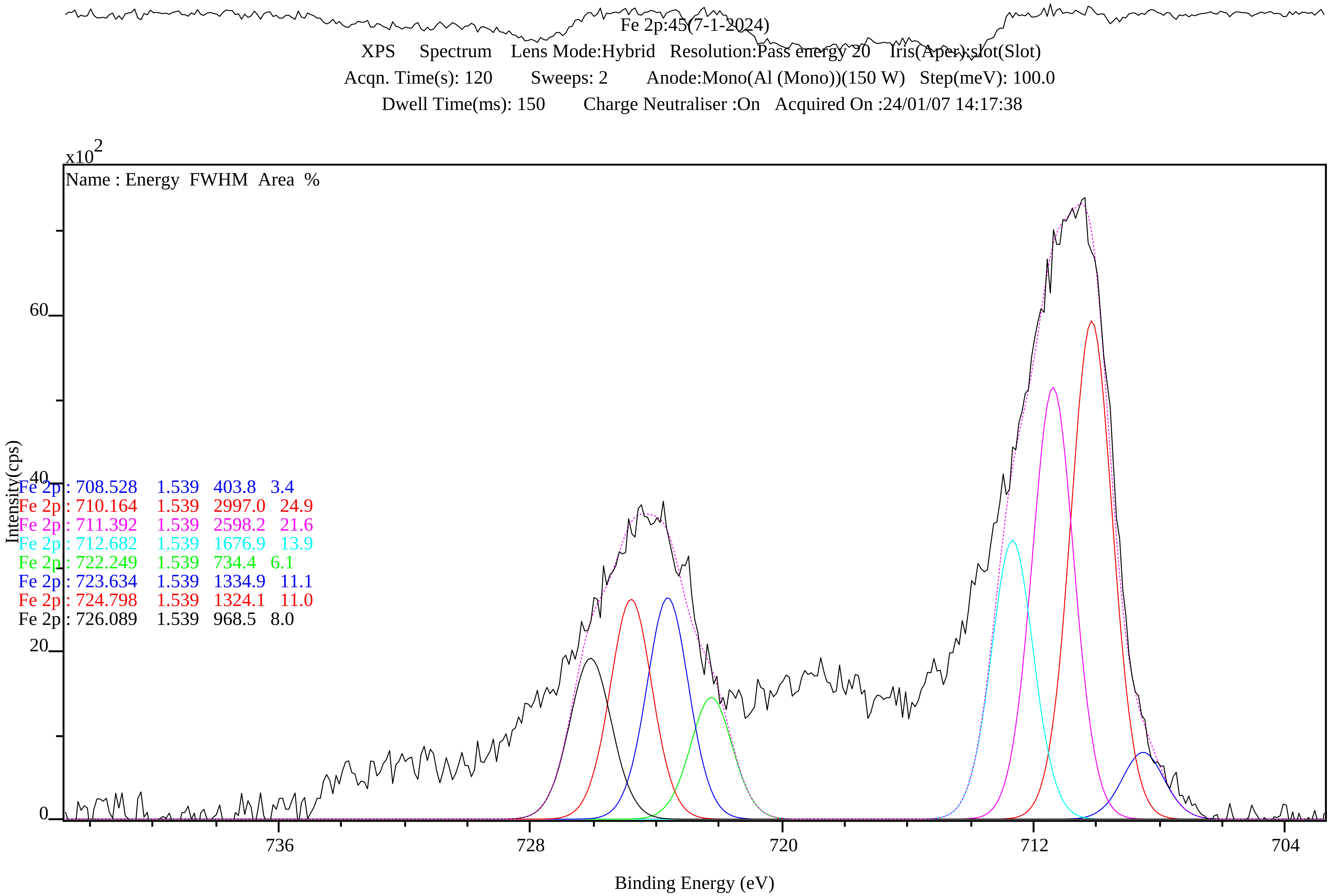

Fe 2p:45(7-1-2024)
XPS Spectrum Lens Mode:Hybrid Resolution:Pass energy 20 Iris(Aper):slot(Slot)
Acqn. Time(s): 120 Sweeps: 2 Anode:Mono(Al (Mono))(150 W) Step(meV): 100.0
Dwell Time(ms): 150 Charge Neutraliser :On Acquired On :24/01/07 14:17:38
x10^2
Name : Energy FWHM Area %
Fe 2p : 708.528 1.539 403.8 3.4
Fe 2p : 710.164 1.539 2997.0 24.9
Fe 2p : 711.392 1.539 2598.2 21.6
Fe 2p : 712.682 1.539 1676.9 13.9
Fe 2p : 722.249 1.539 734.4 6.1
Fe 2p : 723.634 1.539 1334.9 11.1
Fe 2p : 724.798 1.539 1324.1 11.0
Fe 2p : 726.089 1.539 968.5 8.0
Intensity(cps)
60
40
20
0
736
728
720
712
704
Binding Energy (eV)

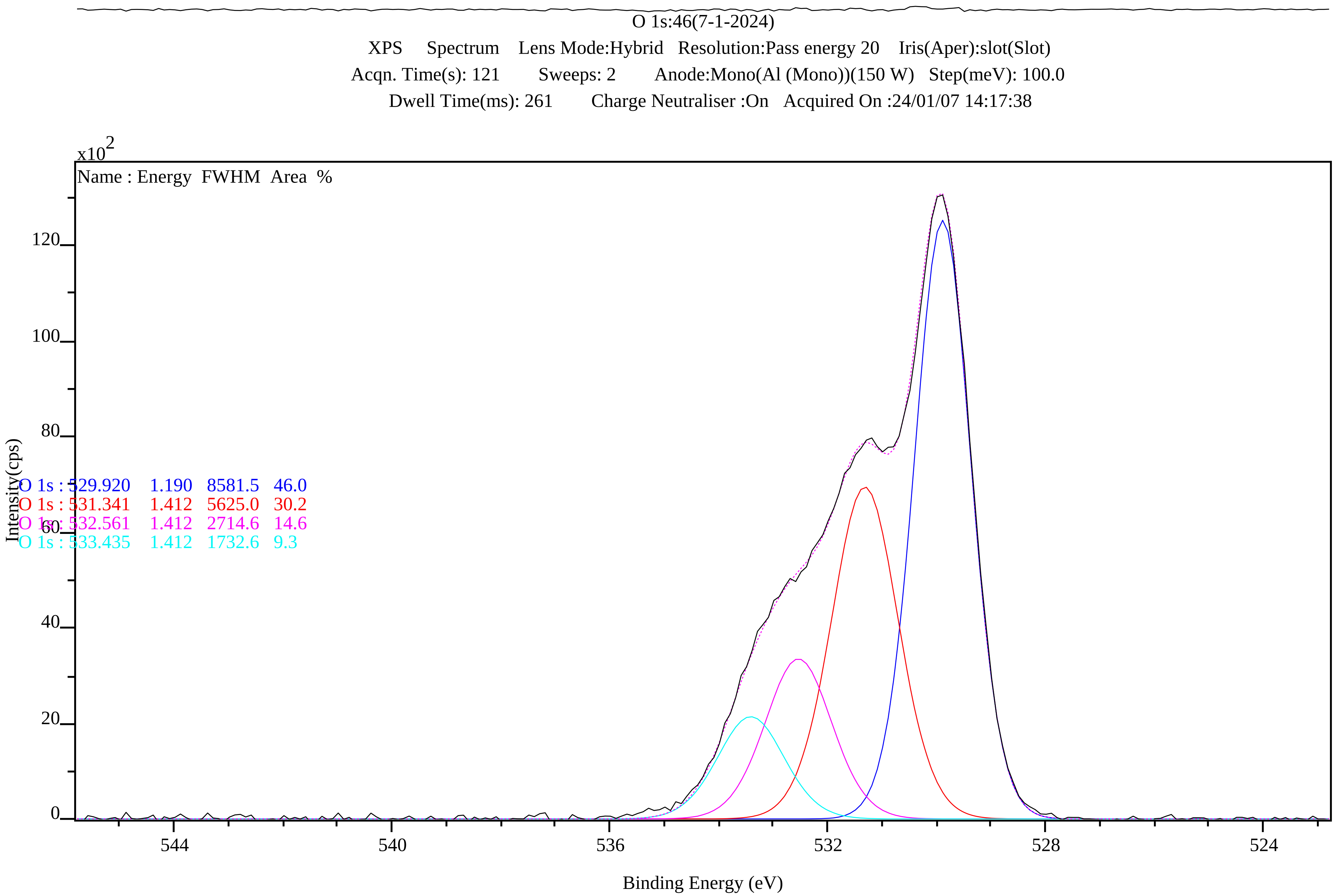

O 1s:46(7-1-2024)
XPS Spectrum Lens Mode:Hybrid Resolution:Pass energy 20 Iris(Aper):slot(Slot)
Acqn. Time(s): 121 Sweeps: 2 Anode:Mono(Al (Mono))(150 W) Step(meV): 100.0
Dwell Time(ms): 261 Charge Neutraliser :On Acquired On :24/01/07 14:17:38
x10^2
Name : Energy FWHM Area %
O 1s : 529.920 1.190 8581.5 46.0
O 1s : 531.341 1.412 5625.0 30.2
O 1s : 532.561 1.412 2714.6 14.6
O 1s : 533.435 1.412 1732.6 9.3
Intensity(cps)
120
100
80
60
40
20
0
544
540
536
532
528
524
Binding Energy (eV)

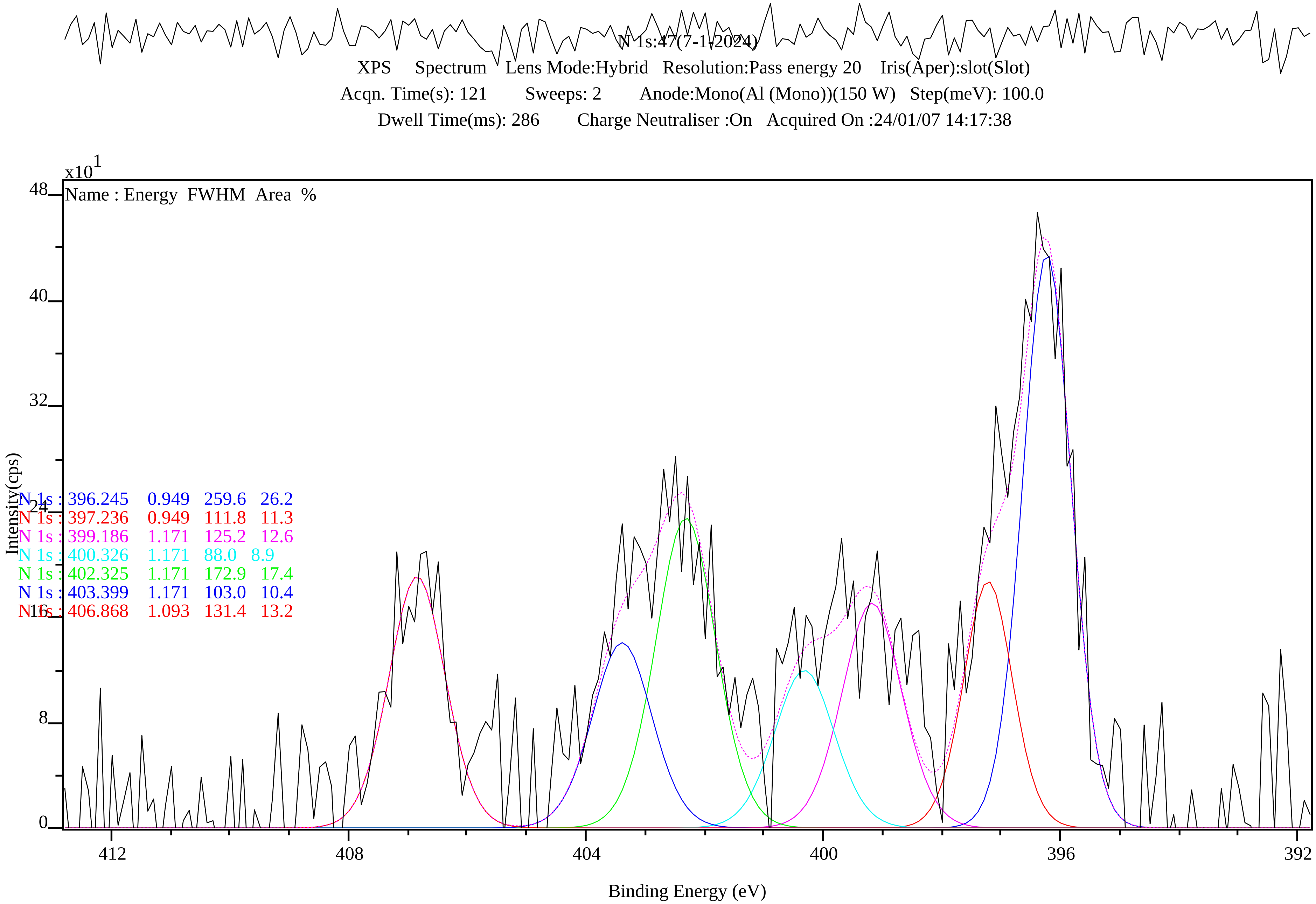
N 1s:47(7-1-2024)
XPS Spectrum Lens Mode:Hybrid Resolution:Pass energy 20 Iris(Aper):slot(Slot)
Acqn. Time(s): 121 Sweeps: 2 Anode:Mono(Al (Mono))(150 W) Step(meV): 100.0
Dwell Time(ms): 286 Charge Neutraliser :On Acquired On :24/01/07 14:17:38
x10^1
Name : Energy FWHM Area %
N 1s : 396.245 0.949 259.6 26.2
N 1s : 397.236 0.949 111.8 11.3
N 1s : 399.186 1.171 125.2 12.6
N 1s : 400.326 1.171 88.0 8.9
N 1s : 402.325 1.171 172.9 17.4
N 1s : 403.399 1.171 103.0 10.4
N 1s : 406.868 1.093 131.4 13.2
Intensity(cps)
48
40
32
24
16
8
0
412
408
404
400
396
392
Binding Energy (eV)

C 1s:48(7-1-2024)

XPS Spectrum Lens Mode:Hybrid Resolution:Pass energy 20 Iris(Aper):slot(Slot)

Acqn. Time(s): 120 Sweeps: 2 Anode:Mono(Al (Mono))(150 W) Step(meV): 100.0

Dwell Time(ms): 250 Charge Neutraliser :On Acquired On :24/01/07 14:17:38

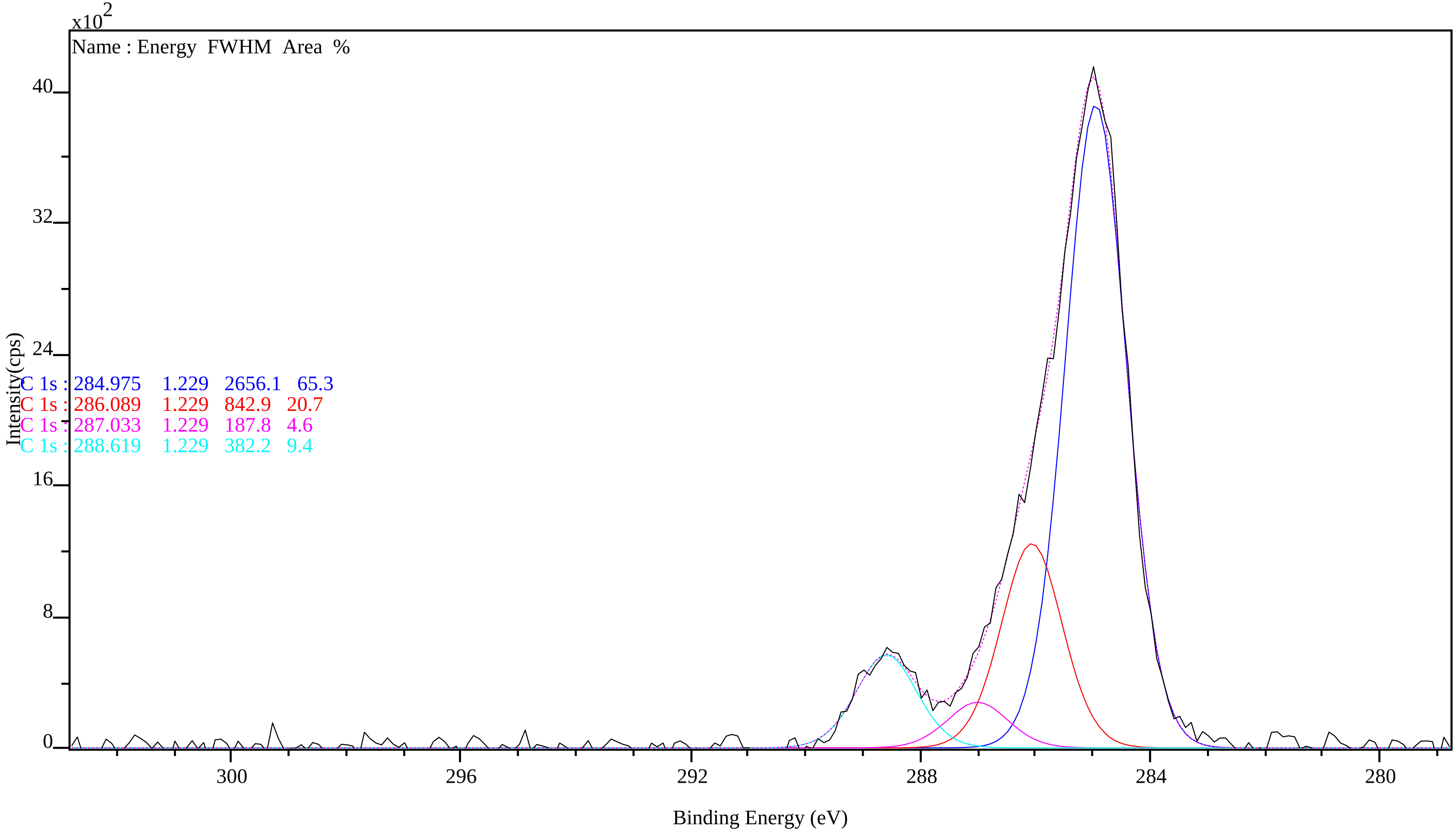

Supplement: Supplementary file 1 [file sensors-24-05466-s001.zip › XPS_7-1-2024/7.Fresh N2 magnet/component.pdf]
