# Supplementary material for: Enhancing the Longevity and Structural Stability of Humidity Sensors: Iron Thin Films with Nitride Bonding Synthesized via Magnetic Field-Assisted Sparking Discharge
Source: Sensors (Basel). 2024 Aug 23;24(17):5466. doi: 10.3390/s24175466 (PMC11397896; doi:10.3390/s24175466)
Supplement: Supplementary file 1 [file sensors-24-05466-s001.zip › XPS_7-1-2024/7.Fresh N2 magnet/report.pdf]

Quantification Report

/C=/data/7-1-2024/7-1-2024.dset Fri Jan 12 13:30:24 2024

State : Angle Name : Position 7

Peak	Type	Position BE (eV)	FWHM (eV)	Raw Area (cps eV)	RSF	Atomic Mass	Atomic Conc %	Mass Conc %
Fe 2p	Reg	711.000	5.324	588695.1	2.957	55.846	26.38	57.45
O 1s	Reg	530.000	4.001	228860.0	0.780	15.999	40.20	25.09
N 1s	Reg	396.000	2.092	7850.0	0.477	14.007	2.32	1.27
C 1s	Reg	285.000	2.457	53930.0	0.278	12.011	28.52	13.36
Si 2p	Reg	103.000	2.402	5980.0	0.328	28.086	2.58	2.83
