# Supplementary material for: Enhancing the Longevity and Structural Stability of Humidity Sensors: Iron Thin Films with Nitride Bonding Synthesized via Magnetic Field-Assisted Sparking Discharge
Source: Sensors (Basel). 2024 Aug 23;24(17):5466. doi: 10.3390/s24175466 (PMC11397896; doi:10.3390/s24175466)
Supplement: Supplementary file 1 [file sensors-24-05466-s001.zip › XPS_7-1-2024/8.Fresh O2 magnet/component.pdf]

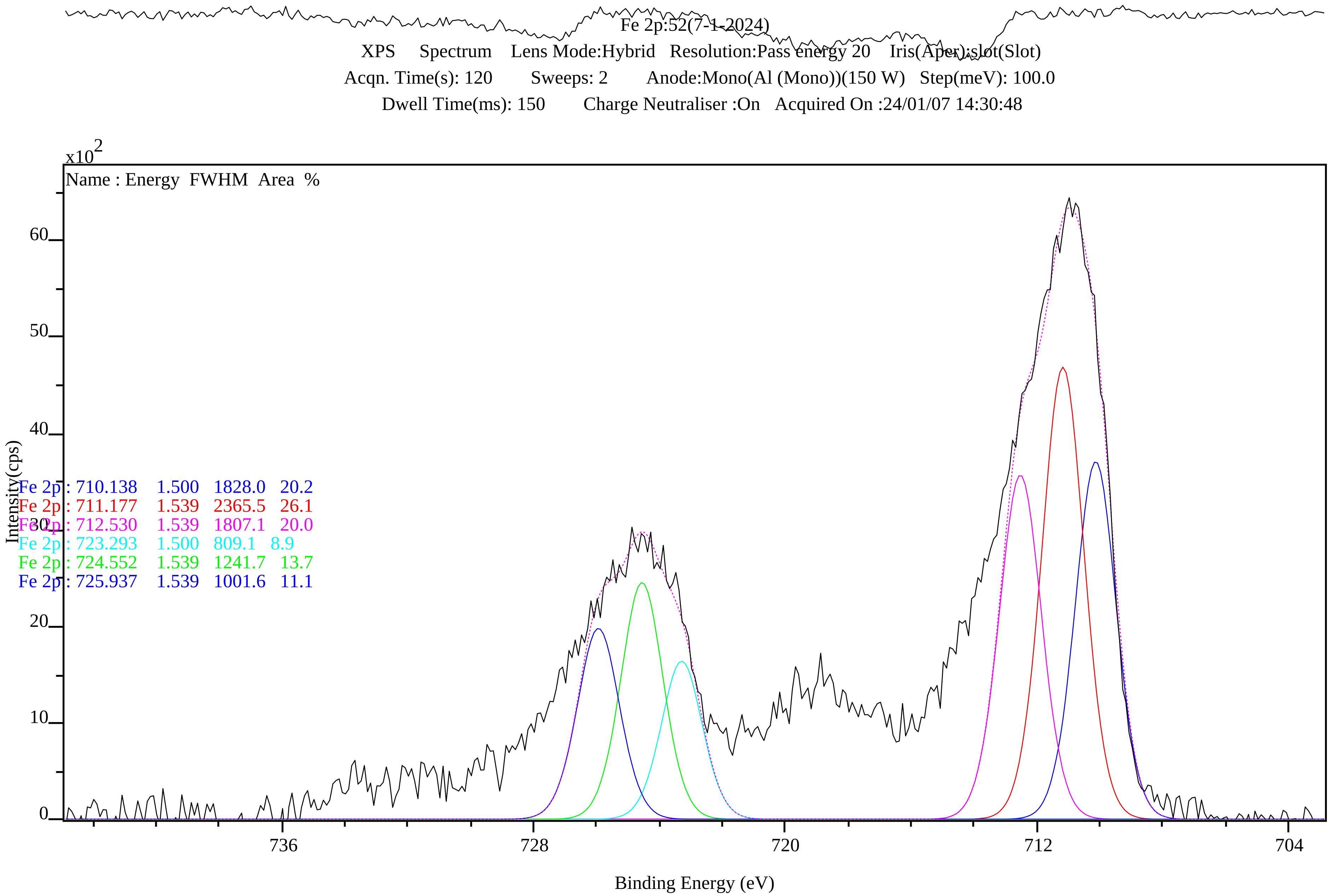
Fe 2p:52(7-1-2024)
XPS Spectrum Lens Mode:Hybrid Resolution:Pass energy 20 Iris(Aper):slot(Slot)
Acqn. Time(s): 120 Sweeps: 2 Anode:Mono(Al (Mono))(150 W) Step(meV): 100.0
Dwell Time(ms): 150 Charge Neutraliser :On Acquired On :24/01/07 14:30:48
x10^2
Name : Energy FWHM Area %
Fe 2p : 710.138 1.500 1828.0 20.2
Fe 2p : 711.177 1.539 2365.5 26.1
Fe 2p : 712.530 1.539 1807.1 20.0
Fe 2p : 723.293 1.500 809.1 8.9
Fe 2p : 724.552 1.539 1241.7 13.7
Fe 2p : 725.937 1.539 1001.6 11.1
Intensity(cps)
0
10
20
30
40
50
60
736
728
720
712
704
Binding Energy (eV)

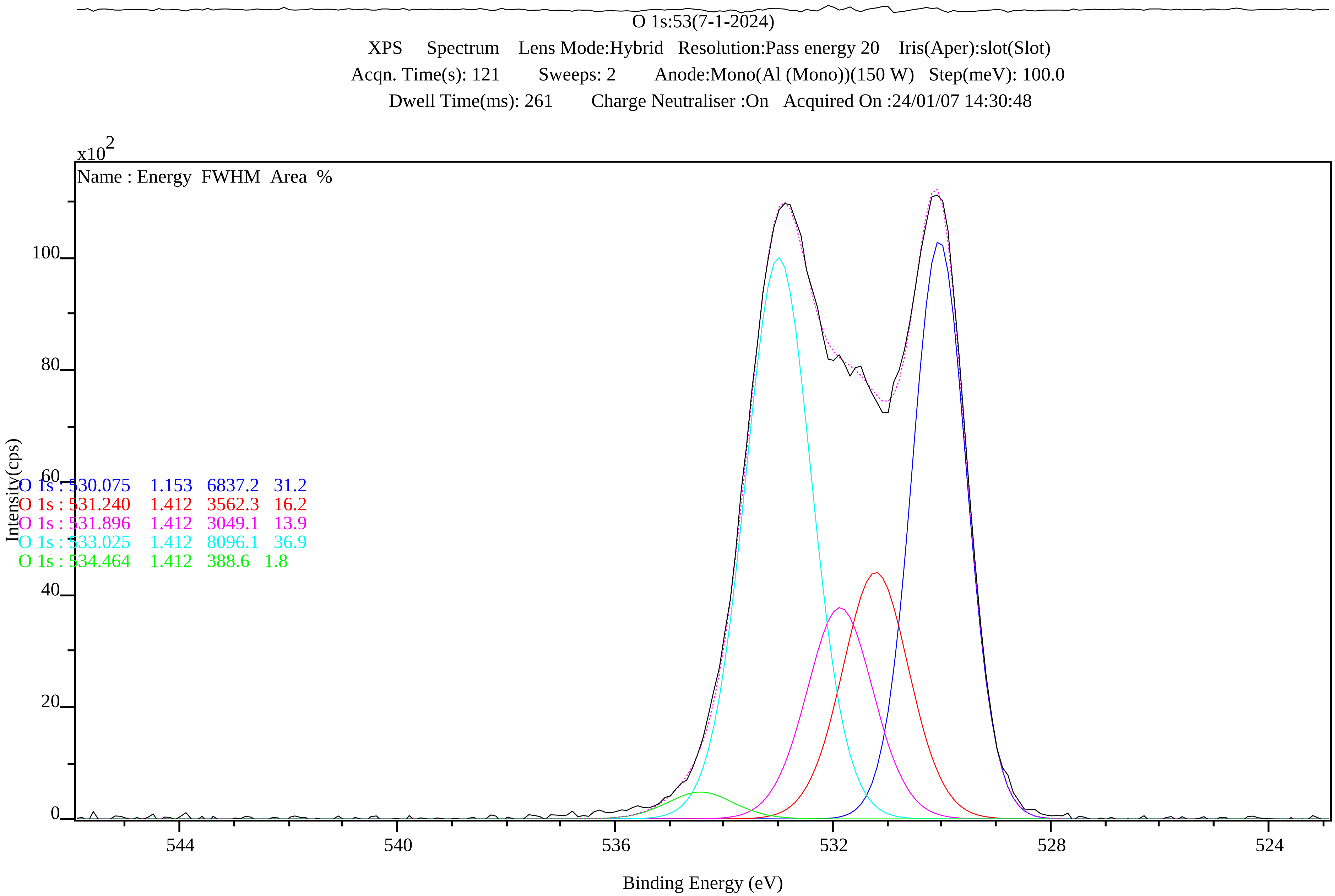
O 1s:53(7-1-2024)
XPS Spectrum Lens Mode:Hybrid Resolution:Pass energy 20 Iris(Aper):slot(Slot)
Acqn. Time(s): 121 Sweeps: 2 Anode:Mono(Al (Mono))(150 W) Step(meV): 100.0
Dwell Time(ms): 261 Charge Neutraliser :On Acquired On :24/01/07 14:30:48
x10^2
Name : Energy FWHM Area %
O 1s : 530.075 1.153 6837.2 31.2
O 1s : 531.240 1.412 3562.3 16.2
O 1s : 531.896 1.412 3049.1 13.9
O 1s : 533.025 1.412 8096.1 36.9
O 1s : 534.464 1.412 388.6 1.8
Intensity(cps)
100
80
60
40
20
0
544
540
536
532
528
524
Binding Energy (eV)

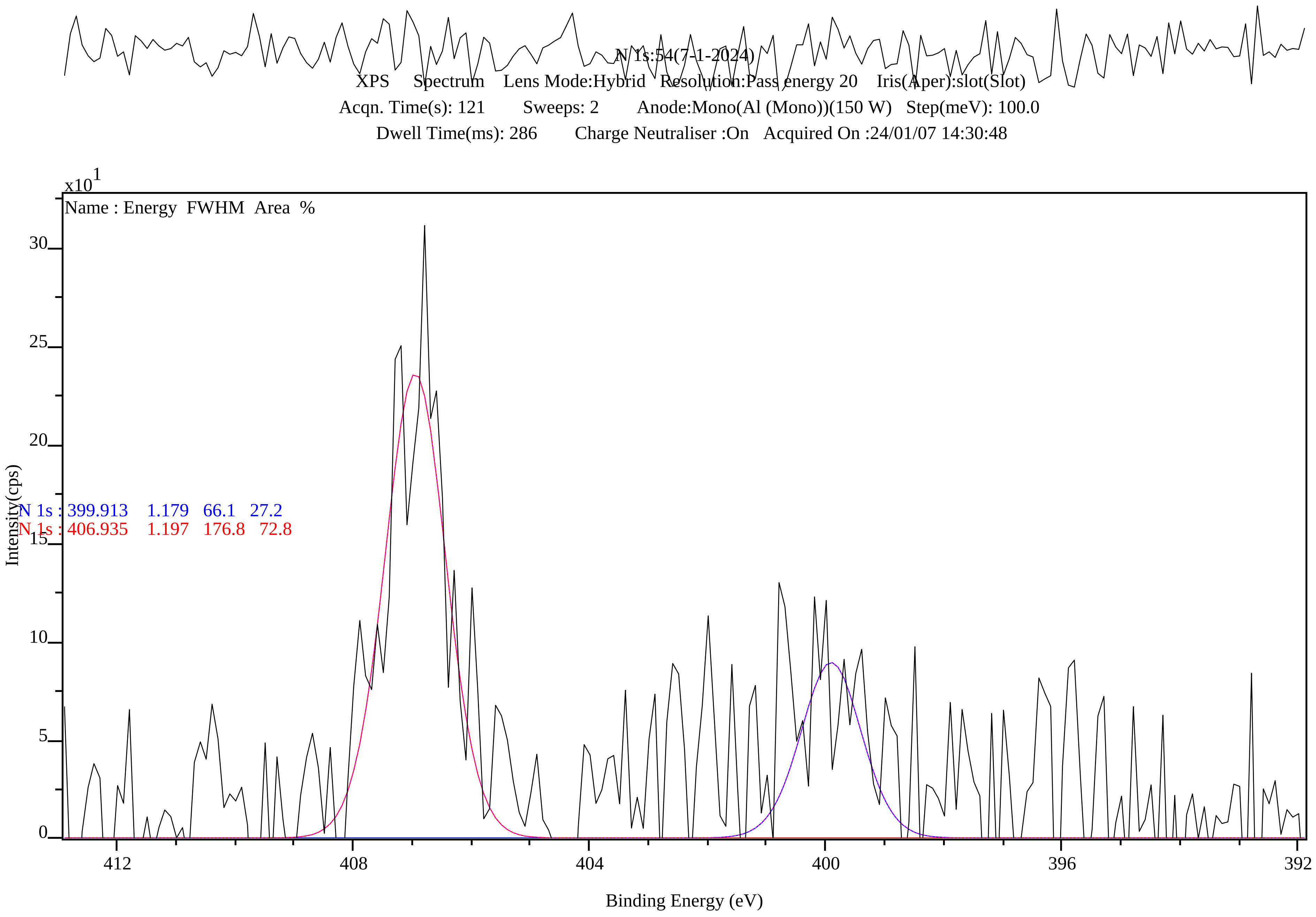

N 1s:54(7-1-2024)
XPS Spectrum Lens Mode:Hybrid Resolution:Pass energy 20 Iris(Aper):slot(Slot)
Acqn. Time(s): 121 Sweeps: 2 Anode:Mono(Al (Mono))(150 W) Step(meV): 100.0
Dwell Time(ms): 286 Charge Neutraliser :On Acquired On :24/01/07 14:30:48
x10^1
Name : Energy FWHM Area %
N 1s : 399.913 1.179 66.1 27.2
N 1s : 406.935 1.197 176.8 72.8
Intensity(cps)
30
25
20
15
10
5
0
412
408
404
400
396
392
Binding Energy (eV)

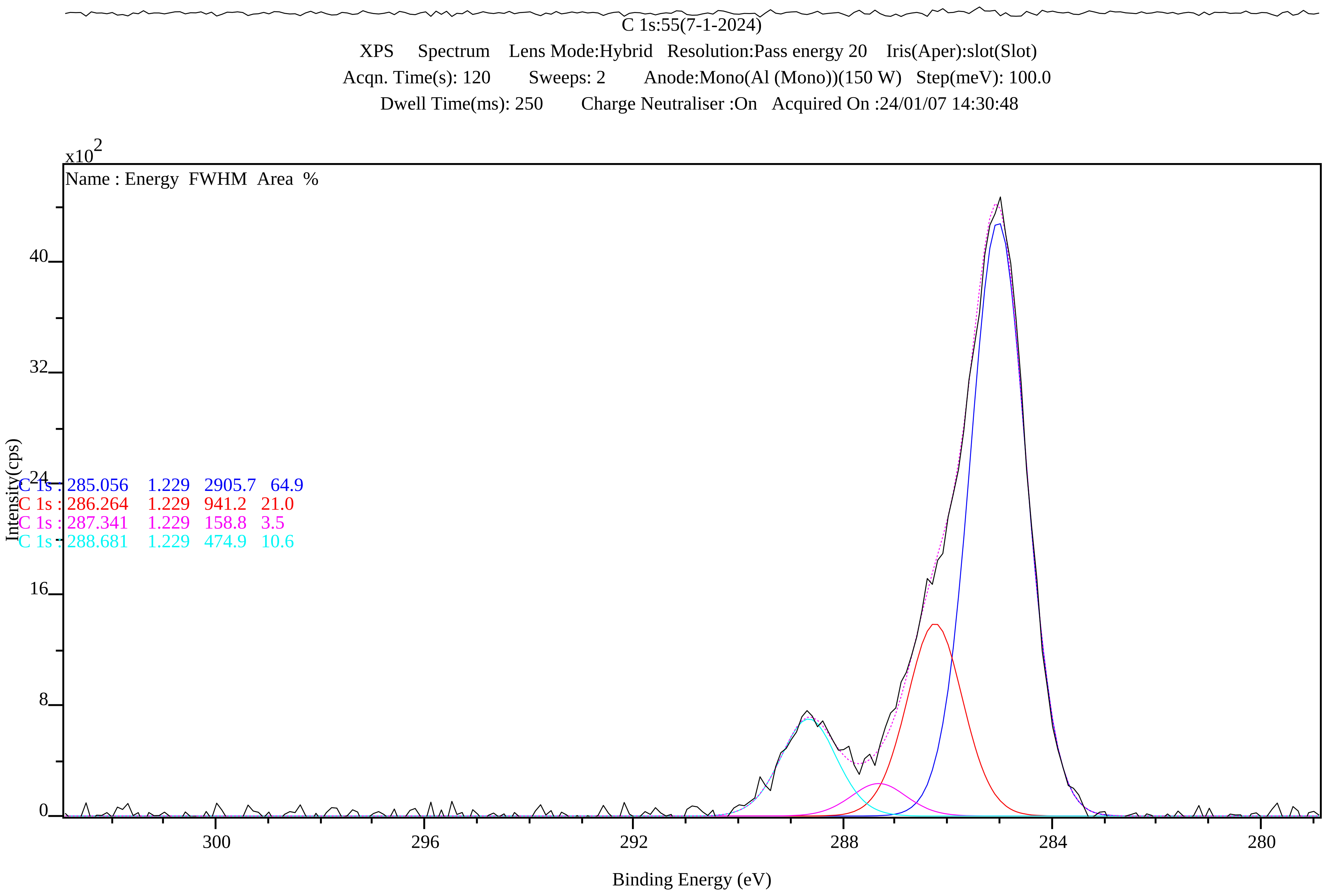
C 1s:55(7-1-2024)
XPS Spectrum Lens Mode:Hybrid Resolution:Pass energy 20 Iris(Aper):slot(Slot)
Acqn. Time(s): 120 Sweeps: 2 Anode:Mono(Al (Mono))(150 W) Step(meV): 100.0
Dwell Time(ms): 250 Charge Neutraliser :On Acquired On :24/01/07 14:30:48
x10^2
Name : Energy FWHM Area %
C 1s : 285.056 1.229 2905.7 64.9
C 1s : 286.264 1.229 941.2 21.0
C 1s : 287.341 1.229 158.8 3.5
C 1s : 288.681 1.229 474.9 10.6
Intensity(cps)
40
32
24
16
8
0
300
296
292
288
284
280
Binding Energy (eV)
