# Supplementary material for: Enhancing the Longevity and Structural Stability of Humidity Sensors: Iron Thin Films with Nitride Bonding Synthesized via Magnetic Field-Assisted Sparking Discharge
Source: Sensors (Basel). 2024 Aug 23;24(17):5466. doi: 10.3390/s24175466 (PMC11397896; doi:10.3390/s24175466)
Supplement: Supplementary file 1 [file sensors-24-05466-s001.zip › XPS_7-1-2024/8.Fresh O2 magnet/report.pdf]

Quantification Report

/C=/data/7-1-2024/7-1-2024.dset Fri Jan 12 13:31:46 2024

State : Angle Name : Position 8

Peak	Type	Position BE (eV)	FWHM (eV)	Raw Area (cps eV)	RSF	Atomic Mass	Atomic Conc %	Mass Conc %
Zn 2p	Reg	1021.000	1.888	11900.0	5.589	65.387	0.24	0.66
Fe 2p	Reg	711.000	5.090	484655.0	2.957	55.846	19.30	45.95
O 1s	Reg	532.000	4.606	277475.0	0.780	15.999	43.30	29.52
N 1s	Reg	407.000	1.888	3970.0	0.477	14.007	1.04	0.62
C 1s	Reg	285.000	2.599	62060.0	0.278	12.011	29.18	14.94
Si 2p	Reg	103.000	2.440	18090.0	0.328	28.086	6.95	8.31
