# Supplementary material for: Enhancing the Longevity and Structural Stability of Humidity Sensors: Iron Thin Films with Nitride Bonding Synthesized via Magnetic Field-Assisted Sparking Discharge
Source: Sensors (Basel). 2024 Aug 23;24(17):5466. doi: 10.3390/s24175466 (PMC11397896; doi:10.3390/s24175466)
Supplement: Supplementary file 1 [file sensors-24-05466-s001.zip › XPS_7-1-2024/8.Fresh O2 magnet/survey.pdf]

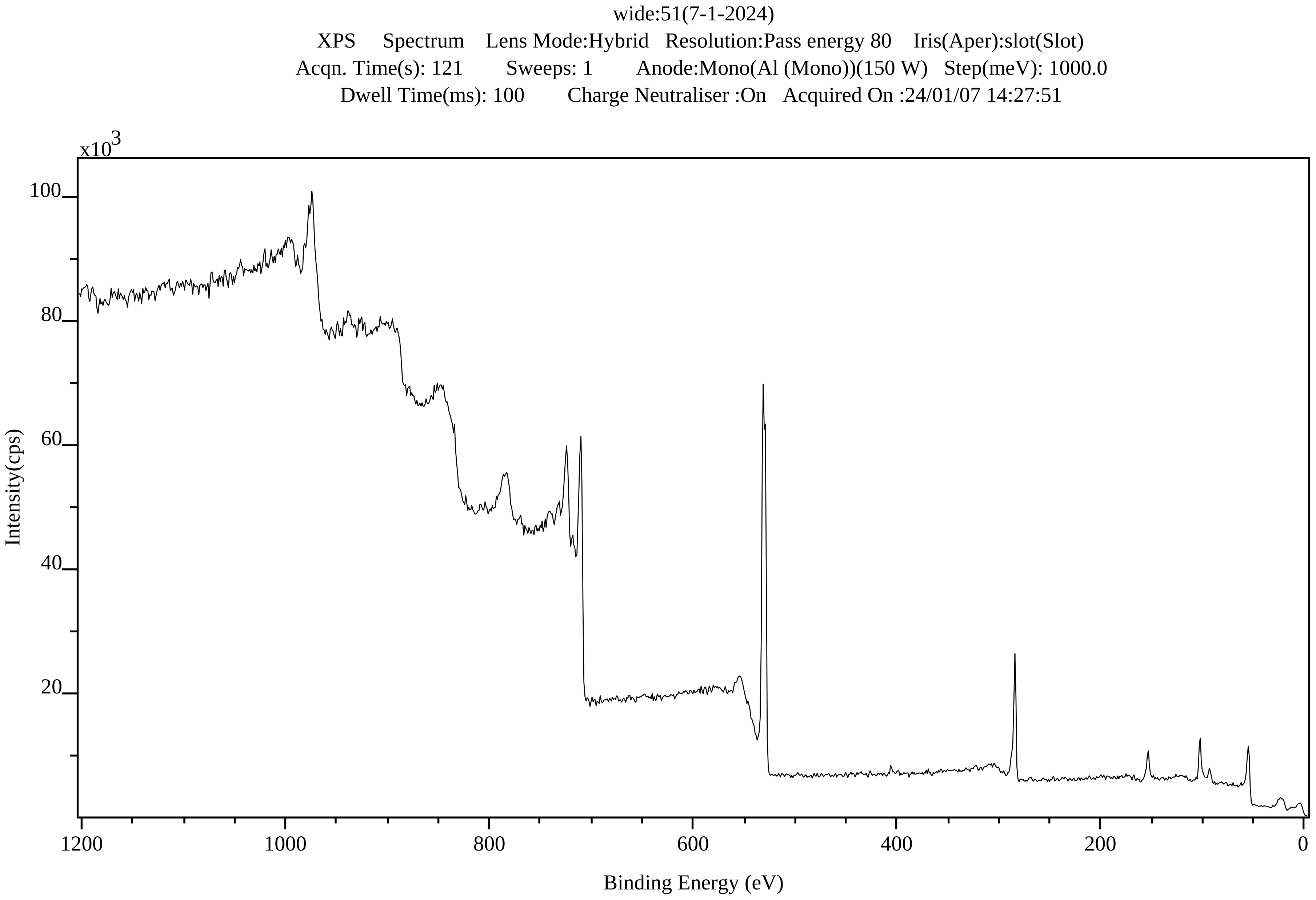
wide:51(7-1-2024)
XPS Spectrum Lens Mode:Hybrid Resolution:Pass energy 80 Iris(Aper):slot(Slot)
Acqn. Time(s): 121 Sweeps: 1 Anode:Mono(Al (Mono))(150 W) Step(meV): 1000.0
Dwell Time(ms): 100 Charge Neutraliser :On Acquired On :24/01/07 14:27:51
x10^3
Intensity(cps)
100
80
60
40
20
1200
1000
800
600
400
200
0
Binding Energy (eV)
